# Mitochondrial Membrane Potential Identifies a Subpopulation of Mesenchymal Progenitor Cells to Promote Angiogenesis and Myocardial Repair

**DOI:** 10.3390/cells11101713

**Published:** 2022-05-22

**Authors:** Xiuchun Li, Xiaoliang Wang, Pan He, Edward Bennett, Erin Haggard, Jianjie Ma, Chuanxi Cai

**Affiliations:** 1 Department of Surgery, Davis Heart and Lung Research Institute, The Ohio State University Wexner Medical Center, Columbus, OH 43210, USA; lxchosu@hotmail.com (X.L.); xiaoliang.wang@osumc.edu (X.W.); erin.haggard@osumc.edu (E.H.); jianjie.ma@osumc.edu (J.M.); 2 Department of Molecular and Cellular Physiology, Albany Medical College, Albany, NY 12208, USA; hpan2015@hotmail.com; 3 Division of Cardiothoracic Surgery, Albany Medical Center, Albany, NY 12208, USA; bennette@mail.amc.edu

**Keywords:** mitochondrial membrane potential, mesenchymal progenitor cells, cytokine, angiogenesis, myocardial infarction, heart failure

## Abstract

Identifying effective donor cells is one of obstacles that limits cell therapy for heart disease. In this study, we sorted a subpopulation of human mesenchymal progenitor cells (hMPCs) from the right atrial appendage using the low mitochondrial membrane potential. Compared to the non-sorted cells, hMPCs hold the capacity for stemness and enrich mesenchymal stem cell markers. The hMPCs display better ability for survival, faster proliferation, less production of reactive oxygen species (ROS), and greater release of cytoprotective cytokines. The hMPCs exhibit decreased expression of senescence genes and increased expression of anti-apoptotic and antioxidant genes. Intramyocardial injection of hMPCs into the infarcted heart resulted in increased left ventricular ejection fraction and reduced cardiac remodeling and infarct size in the group of animals receiving hMPCs. Both in vitro and in vivo studies indicated hMPCs have the potential to differentiate into endothelial cells and smooth muscle cells. Immunohistochemistry staining showed that cell therapy with hMPCs enhances cardiac vascular regeneration and cardiac proliferation, and decreases cardiac cell apoptosis, which is associated with the increased secretion of cytoprotective and pro-angiogenic cytokines. Overall, we discovered a subpopulation of human mesenchymal progenitor cells via their low mitochondrial membrane potential, which might provide an alternative donor cell source for cellular therapy for ischemic heart disease.

## 1. Introduction

Ischemic heart disease is a fatal disorder that involves the permanent loss of myocardium that is replaced by fibrotic scarring, resulting in cardiac dysfunction followed by heart failure [1,2,3]. A variety of therapeutic strategies have been proposed for the treatment of ischemic heart diseases, including heart transplantation for patients at the end stage [1]. Cell-based therapy has generated substantial enthusiasm as a promising treatment for ischemic heart disease [4]. Cardiac progenitor cells (CPCs) are a heterogeneous population of cells that reside throughout the heart, including the ventricles, atria, epicardium, and pericardium [5]. Various approaches have been used to sort and define CPCs. One of the most frequently used approaches is based on the expression of cell surface markers, such as c-kit^+^, Sca-1^+^, SSEA-1^+^, Isl-1^+^, ALDH^br^, Lphn2^+^, side population CPCs, and cardio-sphere derived CPCs [6,7,8,9,10,11,12,13,14,15]. In the past two decades, cell-based therapies have shown the potential to alleviate left ventricular (LV) dysfunction, resulting in the formation of new cardiac tissue, reductions in the infarct size, and attenuation of adverse remodeling following myocardial infarction (MI) in experiments with both small and large animal models, as well as in humans [16,17,18,19,20,21,22,23,24,25,26,27]. There are diverse mechanistic explanations for the heart function benefits observed in therapeutics of cardiac progenitor cells or other cell types, including inflammatory modulation; transdifferentiation into endothelial cells, smooth muscle cells, or cardiomyocytes; exosome release; and paracrine stimulation of angiogenesis or endogenous cardiac progenitors [17,28,29,30,31,32,33,34]. The paracrine mechanism is widely accepted as being primarily responsible for the improved cardiac function observed with the administration of most cell types [30,35].

However, there remain major challenges for adult progenitor cell therapy for ischemic heart diseases, including the poor potency of transplanted cells, low engraftment rates, and retention of the administered cells, as well as the limited donor cell sources for cellular therapeutics [35]. Furthermore, the effects of adult cardiac progenitor cells on myocardial regeneration have been controversial, since multiple groups have mapped the fate of endogenous c-kit^+^ or Sca-1^+^ cardiac progenitor cells with Cre-loxP mouse line systems [36,37,38,39]. However, other investigators argue that there is a limitation on the genetic tracking strategy of c-kit^+^ or Sca-1^+^ cardiac progenitor cells [40]. Therefore, investigators from different laboratories replicating the original CPCs’ findings are faced with challenges in the fields of cardiac regeneration and therapeutics. Further research efforts should focus on developing new strategies to gain more knowledge of cardiac progenitor cells. 

Most of the previously studied CPCs were sorted with special cell surface markers. However, it was reported that low mitochondrial membrane potential (Δψ_m_^low^) T cells show long-term survival, decreased oxidative stress, and superior antitumor activity [41]. The Δψ_m_^low^ hematopoietic stem cells also have the capacity to maintain stemness for cell therapy [41]. In the present study, using the low mitochondrial membrane potential, we sorted a subpopulation of progenitor cells that express mesenchymal stem cell markers and hold the capacity to differentiate into both endothelial cells and smooth muscle cells, named human mesenchymal progenitor cells (hMPCs). Most importantly, intramyocardial transplantation of hMPCs can promote angiogenesis and myocardial repair in an immune-deficient murine MI model.

## 2. Materials and Methods

### 2.1. Human Heart Tissue Collection and Δψ_m_^low^-hMPCs Isolation

The protocol for collecting human samples in this study was approved by the Institutional Committee on Research in Albany Medical College (IRB#3728), and written informed consent was obtained from the patients. Human right atrial appendage (RAA) tissues (age from 42 to 74 years old) were collected and enzymatically digested to establish human cardiac cell lines and defined as RAACs (Supplementary Table S1). We sorted human cardiac cells based on mitochondrial membrane potential (Δψ_m_) and collected ~3% of these cells with the lowest Δψ_m_ that were named as Δψ_m_^low^-hMPCs (abbreviated as hMPCs if not specified). To avoid replicative cell senescence, passage 3 to 10 of hMPCs were used for in vitro experiments, while passage 4 to 6 of hMPCs were used for in vivo experiments. Methodological details are provided in the Supplementary Materials online.

### 2.2. Murine Model of Acute Myocardial Infarction and Intramyocardial Cell Delivery

The in vivo experiments were performed following the National Institutes of Health Guidelines on the Use of Laboratory Animals and were approved by the Albany Medical College Committee on Animal Care. Breeding pairs of NOD.Cg-*Prkdc*^scid^*/Il2rg*^tm1Wjl^*/*SzJ (NSG, Strain #:005557) male and female mice (age 10–12 weeks) were purchased from The Jackson Laboratory (Bar Harbor, ME, USA). The procedure for MI surgery and human cell transplantation via intramyocardial injection was similar to that used in our previous studies of cell transplantation [42]. Briefly, the left anterior descending (LAD) coronary artery was tied for 60 min, followed by reperfusion. RAACs, hMPCs (5 × 10^5^ cells in 40 µL), or an equivalent volume of vehicle were injected intramyocardially using a 30 gauge needle at 45 min after reperfusion. Mice were then allowed to recover and monitored for 39 days until euthanasia and tissue collection. In this study, ~90% of mice survived within 24 h after myocardial surgery. Over 85% of mice survived at 35 days post-surgery. Methodological details are provided in the Supplementary Materials online.

### 2.3. Flow Cytometry

To characterize the expression of cell surface markers for live cells, hMPCs were detached with 0.25% Trypsin-EDTA solution, washed 3 times with PBS, and incubated with antibodies for different cell surface markers at 4 °C for 10 min. FACS analysis was performed using the Guava EasyCyte™ System. Cellular ROS production was measured using a Cellular ROS Detection Assay Kit (Abcam: ab113851, Waltham, MA, USA). The intensity of red fluorescence (ROS) was detected using a Guava EasyCyte™ System. The protocol for mitochondrial membrane potential measurement was exactly followed as described by Sukumar et al. [41]. The intensity of TMRM fluorescence was measured using the Guava EasyCyte™ System. Cell apoptosis was investigated via dual staining with Alexa Fluor 488-Annexin V and propidium iodide (PI) (Invitrogen: V13241, Waltham, MA, USA). The cell apoptosis assay was performed according to the manufacture protocol described previously [10]. Data were analyzed by GuavaSoft™ Module software (EMD Millipore Corporation, Inc.) (accessed on 9 June 2020). To examine the proliferation of hMPCs and RAACs, FACS analysis with BrdU staining was conducted after incubating cultured cells with 10 µM BrdU for 12 h [10]. Detailed information for antibodies and staining protocols is provided in the Supplementary Materials online.

### 2.4. In Vitro Vascular Cell Differentiation from hMPCs

The hMPCs were plated (about 80% confluence) for 24 h before performing endothelial cell differentiation. The protocol for endothelial cell differentiation was as reported by Dr. Oswald et al. [43]. The efficiency of endothelial differentiation was assessed via FACS analysis after staining with the Von Willebrand factor (vWF) endothelial marker. Additionally, smooth muscle cell (SMC) differentiation procedures were exactly followed according to the manufacturer’s instructions, and the efficiency of smooth muscle differentiation was evaluated via FACS analysis after staining with SMC marker α-smooth muscle actin (α-SMA). Additional details together with protocols for vascular differentiation are provided in the Supplementary Materials online. 

### 2.5. Total RNA Extraction and Quantitative Real-Time Polymerase Chain Reaction (RT-qPCR)

The total RNA for each sample was extracted using the Aurum Total RNA mini kit (Bio-Rad: 732-6820, Hercules, CA, USA). The reverse transcription of tRNA to cDNA was performed using the Bio-Rad iScript cDNA Synthesis kit (Bio-Rad: 170-8891). Real-time qPCR was performed to determine the effects of differentiation of human cytokines library I and II screening (Supplementary Tables S2 and S3) and other relative gene expression levels of RAACs and hMPCs. Samples for real-time qPCR were prepared according to the manufacturer’s instructions in iTaq Universal SYBR Green Supermix (Bio-Rad: 172-5124) and were run with a Bio-Rad CFX384 Real-Time system. Please see the RT-qPCR details in the Supplementary Materials online.

### 2.6. Immunoblotting

Western blotting analysis was performed according to a protocol described previously [10]. The chemiluminescent signals were detected using SuperSignal™ West Pico PLUS Chemiluminescent Substrate (ThermoFisher, Waltham, MA, USA) and imaged using the Image Quant LAS 4000 system (accessed on 8 January 2022). The primary antibodies used in this study are listed in the Supplementary Table S6. The secreted cytokines from hMPCs and RAACs were evaluated using the human cytokine antibody library (RayBiotech: AAH-CYT-6-2, (Peachtree Corners, GA, USA) (Supplementary Table S4). The procedure for antibody the array was conducted according to the manufacturer’s instructions. Please see the Supplementary Materials online for additional information.

### 2.7. Colony Formation Assay

Prior to seeding the cells, the 96-well plate was coated with 0.2% gelatin (Sigma Cat# G9391, Sigma-Aldrich, St. Louis, MO, USA) and incubated at 37 °C for 1 h. RAACs, Δψ_m_^High^-RAACs, and hMPCs were detached with 0.25% Trypsin-EDTA solution and diluted to ~1 cell per 100 μL growth medium. Then, 100 µL of suspended cells was transferred into each well of the 96-well plate. The wells with a single cell at day 0 were marked for continuous monitoring. The culture medium was changed every 3 days; images were taken at day 0, day 3, and day 10 using a ZOETM fluorescent imager (Bio-Rad). The amounts of clone formation among different groups were calculated after 10 days of seeding cells.

### 2.8. Immunohistochemistry Staining

Paraffin-embedded, 5-µm-thick heart sections were deparaffinized in xylene and rehydrated gradually through 100, 95, and 70% ethanol followed by an antigen retrieval procedure. Immunohistochemical staining was performed and confocal images were taken using a Leica confocal laser-scanning microscope (Leica DMI 4000B, Leica Microsystems Inc., Deerfield, IL, USA), which were quantitatively analyzed using Image J software (ImageJ software downloaded from the NIH website (Download (nih.gov, accessed on 6 December 2020)). In all cases, at least six hearts per group were examined. In each heart, we cut serial LV sections and took three sections for quantitative analysis at ~100–120 μm intervals, which were below the ligation site and along the LV longitudinal axis. Detailed staining protocols are described in the Supplementary Materials online.

### 2.9. TUNEL Assay to Assess Cardiac Cell Apoptosis

Terminal deoxynucleotidyltransferase-mediated dUTP nick-end labeling (TUNEL) was performed to detect apoptotic nuclei using the terminal deoxynucleotidyltransferase-mediated in situ fluorescein-conjugated dUTP nick-end labeling technique according to the manufacturer’s protocol (GeneCopoeia, Inc., Rockville, MD, USA). The detailed staining protocol is described in the Supplementary Materials online.

### 2.10. Wheat Germ Agglutinin (WGA) Staining 

To evaluate the cardiac fibrosis after myocardial infarction with or without cell transplantation, wheat germ agglutinin (WGA) (Invitrogen) staining was performed for heart tissue sections among three groups. The procedures were exactly followed according to the manufacturer’s instructions. The detailed staining protocol is described in the Supplementary Materials online.

### 2.11. Echocardiographic and Hemodynamic Analysis

After animals were subjected to anesthesia with 1–2% isoflurane, serial M-mode echocardiographic images were obtained for different groups of mice via the Vevo 3100 imaging platform (VisualSonics, Inc., Toronto, ON, Canada) at baseline (7 days prior to coronary occlusion–reperfusion and cell transplantation) and then 5 days and 35 days after hMPCs administration. At day 39, in vivo cardiac hemodynamic function was evaluated right before euthanasia utilizing the Mikro-Tip^®^ Pressure Volume System (MPVS) and Ultra Foundation System (AD instruments, New South Wales, Australia) with a 1.0 French PVR-1045 micro-tip ultra-miniature pressure–volume (PV) catheter (Millar, Houston, TX, USA). Similar to the echocardiographic study, all hemodynamic data analyses were performed offline by investigators blinded to the treatment. Detailed echocardiographic and hemodynamic analysis protocols are described in the Supplementary Materials online.

### 2.12. Statistical Analysis 

All of the experimental data were analyzed using GraphPad Prism 8.3 software (accessed on 6 March 2022). The standard error of the mean (SEM) is indicated by error bars for each group of data. Data are expressed as means ± SEM. Comparisons were made using Students’ *t*-test when comparing 2 experimental groups. One-way ANOVA followed by Tukey’s multiple comparison test was used to compare data from 3 different groups. Here, *p*-values less than 0.05 were considered statistically significant. 

## 3. Results

### 3.1. Sorting and Characterization of Δψ_m_^low^-hMPC from the Human Right Atrial Appendage

**Figure 1:**
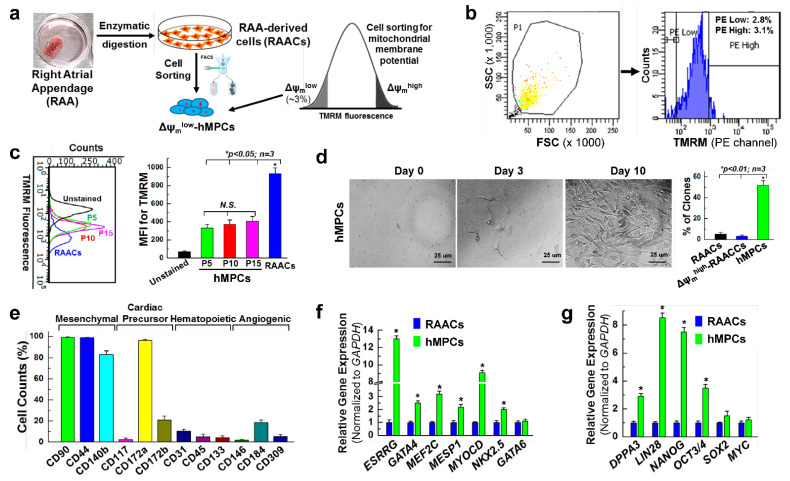
Characterization of Δψmlow-hMPCs isolated from human right atrial appendage. (**a**) Schematic protocol used for isolating RAACs and sorting Δψ_m_^low^-hMPCs (abbreviated as hMPCs) from human right atrial appendage. (**b**) Representative images of sorting hMPCs by flow cytometer, whereby 3% cells with the lowest mitochondrial membrane potential were collected and defined as hMPCs. (**c**) The hMPCs maintained the low mitochondrial membrane potential following a long-term cell expansion for up to 15 passages. Control RAACs were used passage 5. “N.S.” stands for no significant difference. (**d**) Representative images show the clonogenic formation from single RAACs, Δψ_m_^high^-RAACs, and hMPCs after 10 days of cell seeding. The right panel shows the quantitative percentages of colony formation among three groups of cells. (**e**) Quantitative results showing the percentages of mesenchymal, cardiac precursor, hematopoietic, and angiogenic cell surface marker expression in hMPCs. (**f**,**g**) Quantitative PCR analysis showing the relative mRNA expression of cardiac lineage marker genes (**f**) and stem cell lineage marker genes (**g**) in both hMPCs and RAACs. The y-axis represents relative mRNA expression levels of target genes that were normalized to GAPDH as an internal control. Data are means ± SEM. Note: * *p* < 0.05, unpaired Students’ *t*-test, *n* = 3 independent experiments.

To examine whether progenitor cells with lower mitochondria membrane potential have the potential to repair an infarcted heart, we sorted a subpopulation of adult human progenitor cells based on mitochondrial membrane potential (Δψm). Briefly, adult human cardiac cells derived from the human right atrial appendage (RAACs) were subjected to live cell staining with tetramethylrhodamine (TMRM) dye. FACS sorting was conducted to collect the ~3% of cells with the lowest Δψm, which were used for further cell expansion and the following experiments (Figure 1a,b). Based on our results presented below, we defined these cells as human mesenchymal progenitor cells with low mitochondrial membrane potential (Δψmlow-hMPCs, abbreviated as hMPCs below if not specified). The ~3% of RAACs cells with the highest Δψm (Δψmhigh-RAACs) were also collected and served as one of the control cells. To test whether long-term cell culture can change the characteristics of low mitochondrial membrane potential, TMRM dye staining was performed for hMPCs at passage 5, 10, and 15, and the Δψm was measured via FACS analysis. The results showed that hMPCs maintained the low Δψm characteristics along with cells cultured for up to 15 passages (Figure 1c). Single hMPC, RAAC, and Δψmhigh-RAAC were seeded into 96-well plates to check whether these cells have the capacity for stemness. As shown in Figure 1d, 53.8% of single hMPCs can form a colony; however, the rates of colony formation for both RAACs and Δψmhigh-RAACs are less than 10%. 

The expression levels of different stem cell surface markers were examined by FACS analysis of hMPCs after live cell staining with antibodies against individual cell surface markers (Figure 1e). These results showed that hMPCs enriched mesenchymal stem cell markers (99.3% positive for CD90, 99.8% positive for CD44, and 83.2% positive for CD140b) and cardiac precursor markers (96.4% positive for CD172a and 21.1% positive for CD172b). Therefore, we named these cells human mesenchymal progenitor cells (hMPCs). However, about 2.73% of hMPCs are positive for the traditional cardiac stem cell marker, CD117 (c-kit), indicating that most of these cells are c-kit-negative cells. The cell surface marker staining also indicated that hMPCs contained relatively low percentages of hematopoietic stem cells and angiogenic progenitor cells based on the staining with hematopoietic makers (CD31, 10.0%) and angiogenic markers (CD184, 18.5%) (Figure 1e, Supplementary Figures S1 and S2). The qPCR results showed that the expression rates of cardiac lineage transcripts genes, including ESRRG, GATA4, MEF2C, MESP1, MYOCD, and NKX2.5, are significantly higher in hMPCs compared to RAACs (Figure 1f). Meanwhile, the real-time qPCR analysis results indicated that hMPCs have remarkably higher levels of expression of the pluripotent stem cell marker genes, including DPPA3, LIN28, NANOG, and OCT3/4 (Figure 1g).

### 3.2. hMPCs Exhibits Better Cellular Functions

**Figure 2:**
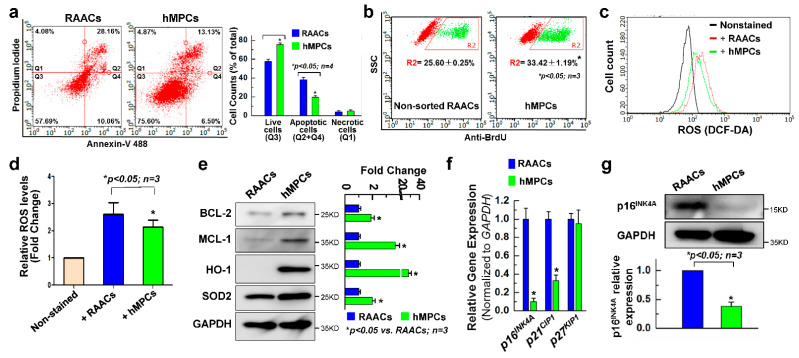
The hMPCs exhibit better reparative capacity. (**a**) Apoptotic assay with the double staining of Annexin V and propidium iodide (PI) in hMPCs and RAACs following the oxidative stress induced by H_2_O_2_. (**b**) FACS analysis for the proliferation assay following the incorporation and staining with BrdU in hMPCs and RAACs. (**c**) Reactive oxygen species (ROS) measurements for hMPCs and RAACs after staining with 2′,7′-dichlorofluorescin diacetate (DCF-DA) dye. (**d**) Quantitative analysis of ROS levels in hMPCs and RAACs as shown in (**c**). The y-axis represents the relative fold changes of ROS levels. (**e**) Western blots for the expression anti-apoptotic proteins (BCL-2 and MCL-1) and anti-oxidative proteins (HO-1 and SOD2) in hMPCs and RAACs. (**f**) Quantitative PCR analysis of the mRNA expression of senescence marker genes (p16^INk4a^, p21^CIP1^, and p27^KIP1^) in hMPCs and RAACs. The y-axis represents the relative mRNA expression of target genes normalized to GAPDH. (**g**) Western blots confirmed the lower protein expression of senescence marker p16^INK4a^ in hMPCs versus RAACs. Data are means ± SEM. Note: * *p* < 0.05, unpaired Student’s *t*-test, *n* ≥ 3 independent experiments.

Poor donor cell survival is one of the major obstacles for in vivo cellular therapy of adult progenitor cells [35]. Here, we evaluated whether hMPCs have a better survival capacity compared with non-sorted RAACs. First, flow cytometry analysis was performed for both hMPCs and RAACs after being stained with nnexin V and propidium iodide (PI) to examine the survival ability against oxidative stress induced by H_2_O_2_ (2 mM for 1.5 h). It was observed that hMPCs exhibited a significant increase in the total number of live cells (75.5 ± 1.23%) compared to RAACs (57.7 ± 2.23%), and the number of apoptotic cells decreased by about 19% for hMPCs (Figure 2a). Additionally, cell proliferation was assessed for both hMPCs and RAACs via FACS analysis with the staining of bromodeoxyuridine/5-bromo-2′-deoxyuridine (BrdU) following incubation of cells with BrdU for 12 h. As shown in Figure 2b, hMPCs exhibited significantly increased BrdU+ cells compared to RAACs (33.4 ± 1.19% vs. 25.6 ± 0.25%, respectively). To examine the production levels of ROS between hMPCs and RAACs, FACS analysis was conducted following staining cells with DCF-DA fluorescence dye. Interestingly, the ROS levels were significantly decreased in hMPCs (Figure 2c,d). To understand the underlying mechanism of cell survival and decreased production of ROS for hMPCs, Western blots were performed, which showed significantly increased protein expression of anti-apoptotic proteins BCL2 and MCL-1 and antioxidant proteins HO-1 and SOD2 in hMPCs compared to RAACs (Figure 2e). The real-time qPCR results showed that hMPCs expressed significantly lower levels of senescent genes p16INK4A, p21CIP1, and p27KIP1 compared to the unsorted RAACs (Figure 2f). Western blots confirmed the lower expression levels of p16INK4A protein in hMPCs (Figure 2g), indicating the hMPCs exhibit a younger phenotype in comparison with RAACs. Taken together, these results demonstrate that hMPCs have a superior capacity for cell survival and proliferation and produce less ROS compared to unsorted RAACs.

### 3.3. hMPCs Release Various Cytoprotective and Pro-Angiogenesis Cytokines

**Figure 3:**
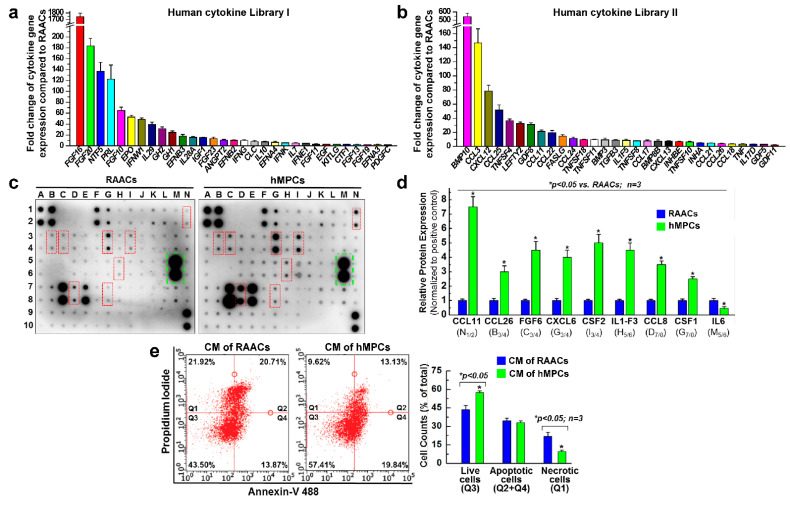
The hMPCs release more cytoprotective and angiogenic cytokines. (**a**,**b**) The qPCR analysis was conducted using a human cytokine gene array with the human cytokine primer libraries I (**a**) and II (**b**) to evaluate the cytokine gene expression levels in hMPCs versus RAACs. (**c**) Western blotting with human cytokine antibody array was performed to assess the released cytokines in hMPCs and RAACs. (**d**) Quantitative analysis of cytokine protein in hMPCs and RAACs. The y-axis represents fold changes of relative protein expression levels of target cytokines in hMPCs versus RAACs. (**e**) Apoptotic assay with the double staining of Annexin V and propidium iodide (PI) for RAACs that were cultured with conditioned medium (CM) collected from hMPCs and RAACs for 48 h. Quantitative analysis is shown in the right panel. The y-axis represents percentage of cell counts to total cells. Data are means ± SEM. Note: * *p* < 0.05, unpaired students’ *t*-test, *n* = 3 independent experiments.

The qPCR analysis with two sets of human cytokine libraries (Supplementary Tables S2 and S3) using a 96-well format was conducted to evaluate the expression of different cytokines in both hMPCs and RAACs. Compared with RAACs, hMPCs exhibited significantly higher expression levels of various cytoprotective and pro-angiogenesis cytokines. More than 60 different cytokines display higher expression levels in hMPCs, such as FGF10/16/20/23, EPO, BMP10, IGF1, EGF, CCL3, CCL25, PRL, GDF8, and others (Figure 3a,b). Furthermore, a human cytokine antibody library array (Supplementary Table S4) was used to assess secreted cytokines by blotting the conditioned medium (CM) collected from both hMPCs and RAACs. The results showed that hMPCs exhibit significant increases in the secretion of several cytokines, including CCL11/26, FGF6, CXCL6, CSF2, IL-F3, CCL8, and CSF1 (Figure 3c,d). These data demonstrate that compared with RAACs, hMPCs secrete more cytoprotective cytokines that might be responsible for promoting cell survival under H_2_O_2_ oxidative stress conditions. Furthermore, to evaluate the effects of secreted cytokines of hMPCs on cell survival, we tested the beneficial effect of the conditioned medium in promoting cell survival capacity. Briefly, the conditioned medium was collected from both hMPCs and RAACs after culturing these cells for 48 h. Conditioned medium samples were then used to culture RAACs for 48 h, followed by treatment with 2 mM H_2_O_2_ for 1.5 h. FACS analysis with double staining of Annexin V and PI demonstrated that conditioned medium from hMPCs promotes the survival of RAACs against H_2_O_2_-induced cell apoptosis, as indicated by a ~14% increase in survival cells (Figure 3e). Taken together, hMPCs prevent H_2_O_2_ stress-induced injury associated with the secretion of cytoprotective cytokines.

### 3.4. Transplantation of hMPCs into Injured Heart Improves Cardiac Structure and Function in an Immunodeficient Mouse MI Model

**Figure 4:**
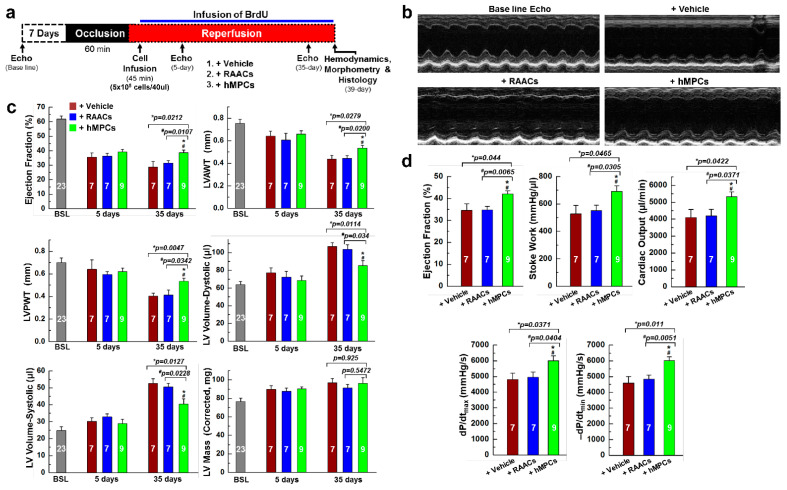
Transplantation of hMPCs into infarcted mouse heart improves cardiac function and structure. (**a**) Schematic protocol for the murine model of acute myocardial infarction and cellular therapy. (**b**) Representative images of M-mode echocardiograms (Echo) at the baseline and at 35 days among three groups of animals with the transplantation of vehicle, RAACs, and hMPCs. (**c**) Echocardiographic parameters for male mice, including ejection fraction (EF), left ventricle anterior wall thickness (LVAWT), left ventricle posterior wall thickness (LVPWT), LV volume—dystonic, LV volume—systolic, and LV mass among the groups of vehicle (*n* = 7), RAACs (*n* = 7) and hMPCs (*n* = 9) at 5 days and 35 days after cell transplantation. “BSL” stands for “baseline”, as shown in (**a**). (**d**) Hemodynamic parameters for the male mice among three groups, including EF, stoke volume, dP/dt_max_, dP/dt_min_, and cardiac output at 39 days after cell transplantation. Data are means ± SEM. Note: * *p* < 0.05 versus Vehicle group, ^#^ *p* < 0.05 versus RAAC group, as calculated using one-way ANOVA followed by Tukey’s multiple comparison test.

To examine the therapeutic efficiency of hMPCs for ischemic heart disease, the immunodeficient NOD acid gamma (NSG) mouse was used to induce myocardial infarction (MI) with 1 h ischemia followed by reperfusion (I/R). The hMPCs were intramyocardially injected into 4 different sites around the risk area immediately after 45 min of reperfusion (Supplementary Figure S3a). The echocardiographic images were collected 7 days prior to, 5 days after, and 35 days after cell transplantation. Hemodynamic studies with pressure–volume (PV) loop measurements were performed right before collecting the heart tissue for immunohistochemistry studies (Figure 4a). During the echocardiographic imaging, the heart rates were maintained stably at similar levels among different groups. Data for the heart rates exhibit no significant changes at 5 days or 35 days after cardiac surgery among the different groups (Supplementary Figure S3b,c). Representative images of M-mode echocardiograms showed that mice receiving hMPCs had preserved cardiac function compared with the groups of mice receiving vehicle and RAACs (Figure 4b,c). For male mice, the left ventricle ejection fractions (LV-EF) were similar among the three groups (vehicle, RAACs, and hMPCs) at 5 days after MI surgery. However, the 5-day LV-EF rates in all three groups were significantly decreased compared with the baseline LV-EF measured prior to the I/R surgery, indicating the severity of LV dysfunction after MI (Figure 4c). It was observed that implantation of hMPCs into the heart preserves the heart function at 35 days after I/R-induced MI, as indicated by the higher EF compared to vehicle or RAAC groups (Figure 4c). Meanwhile, the structural parameters (LVAWT: LV anterior wall thickness; LVPWT: LV posterior wall thickness; LV diastolic and systolic volume) of LV remodeling were similar among the three groups at baseline and 5 days after MI (Figure 4c). However, at 35 days after cell transplantation, both the LV posterior and anterior wall thicknesses (LVAWT, LVPWT) were significantly larger in the hMPC group than that in vehicle and RAAC groups. Furthermore, the LV diastolic and systolic volumes were considerably smaller in the hMPC group than that in vehicle and RAAC groups (Figure 4c). Notably, the results from the hemodynamic analysis were consistent with the echocardiographic data, as indicated by the PV-loop parameters, including increased LVEF, increased stroke work, dP/dtmax, dP/dtmin, and cardiac output values in the hMPC group (Figure 4d). These results suggest that the transplantation of hMPCs exhibits significant beneficial effects in preserving heart function and preventing cardiac remodeling against I/R-induced myocardial injury.

We also assessed the therapeutic efficiency of hMPCs in the MI model of female mice. As shown in Supplementary Figure S4a, improved cardiac structure and preserved cardiac function were observed in the hMPC group for female mice, as indicated by increased LV-EF, LVAWT, and LVPWT rates and decreased LV dystonic volume and systolic volume rates. The hemodynamic analysis results were consistent with echocardiographic data in the female mice (Supplementary Figure S4b). Taken together, our results demonstrated that implantation of hMPCs into infarcted mouse heart preserves cardiac function and suppress left ventricle remodeling in the MI models of both male and female mice.

### 3.5. Transplantation of hMPCs Reduces the Scar Size, Inhibits Cardiac Cell Apoptosis, and Promotes Endogenous Cardiac Regeneration

**Figure 5:**
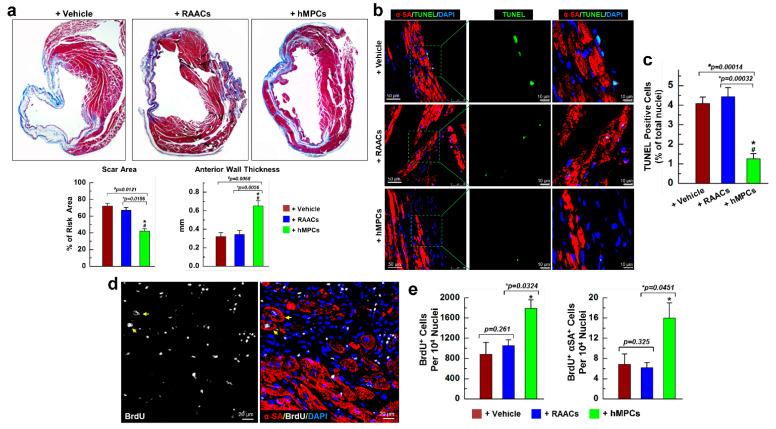
Transplantation of hMPCs reduces the infarct size and cardiac cell apoptosis and promotes endogenous cardiac regeneration. (**a**) Representative images of heart section with Masson’s trichrome staining and the quantitative analysis for the percentage of scar area over the risk area of the left ventricle, as well as the anterior wall thickness of left ventricle. (**b**) Representative confocal images for the staining of terminal deoxynucleotidyl transferase dUTP nick-end labeling (TUNEL) in risk area of infarcted mouse hearts. Green indicates the TUNEL-positive cells, red indicates the αSA-positive cells, and blue indicates the DAPI-positive nuclei. (**c**) Quantitative results for TUNEL^+^ cells. The y-axis represents the percentage of TUNEL^+^ cells over total nuclei. (**d**) Representative confocal images for heart section with BrdU and α-sarcomeric actinin staining. White color indicates the BrdU-positive proliferating cells, which are newly formed cardiac cells. Red color indicates the α-sarcomeric actinin (αSA)-positive cardiomyocytes. Double-positive cells (BrdU^+^αSA^+^) are newly formed or proliferated cardiomyocytes. (**e**) Quantitative results for BrdU^+^ (left) and BrdU^+^αSA^+^ (right). The y-axis stands for the number of BrdU^+^ or BrdU^+^αSA^+^ cells per 10^4^ nuclei. Note: *n* = 6 mice per group, 20 images/slide; data are means ± SEM; * *p* < 0.05 versus Vehicle group, ^#^ *p* < 0.05 versus RAAC group, as calculated by one-way ANOVA followed by Tukey’s multiple comparison test.

LV remodeling was also evaluated using Masson’s trichrome staining. As shown in Figure 5a, a reduction in scar size and an increase in anterior wall thickness were observed in the hearts of animals that received hMPCs compared to in the vehicle and RAAC groups. Furthermore, WGA staining was conducted to detect cardiac fibrosis, which showed that cardiac fibrosis was significantly decreased in the hMPC group compared to vehicle and RAAC groups (ratio of fibrosis: 5.34 ± 0.56% (hMPCs) versus 13.96 ± 1.66% (vehicle) or 14.68 ± 1.46% (RAACs), respectively) (Supplementary Figure S5a,b). 

Meanwhile, TUNEL staining was conducted to assess cell apoptosis in the risk area of the infarcted heart. A significant reduced number of apoptotic cells was observed in the hMPC group compared to vehicle and RAAC groups (Figure 5b,c), suggesting that transplantation of hMPCs prevents cardiac cell apoptosis after MI.

To understand endogenous cardiac regeneration after transplantation of hMPCs into injured hearts, animals were intraperitoneally injected with BrdU (100 mg/kg body weight) once daily for up to five weeks post-MI. At 39 days, we collected hearts for BrdU immunostaining. Confocal images showed that the number of BrdU^+^ cells was significantly increased in the hMPC group compared to vehicle and RAAC groups, indicating that infusion of hMPCs enhanced the endogenous cardiac proliferation in the in vivo murine MI model (Figure 5d,e). Evidence of newly formed or proliferated cardiomyocytes was indicated by the double-positive staining of α-sarcomeric actin (α-SA) and BrdU. We observed newly formed cardiomyocytes in the risk area, as indicated by the double-positive staining of BrdU and α-SA (BrdU^+^α-SA^+^) in mouse hearts (Figure 5d, yellow arrow), with about 16.0 ± 3.92 cells per 10^4^ nuclei for the group of animals receiving hMPCs. The number of BrdU^+^α-SA^+^ cells increased by about two-fold in the hMPC group compared to the vehicle and RAAC groups (Figure 5e, right panel), indicating increased cardiac regeneration following transplantation of hMPCs in the infarcted heart.

### 3.6. hMPCs can Differentiate into Vascular Cells in Both In Vitro Cell Culture and In Vivo Myocardial Repair Conditions

**Figure 6:**
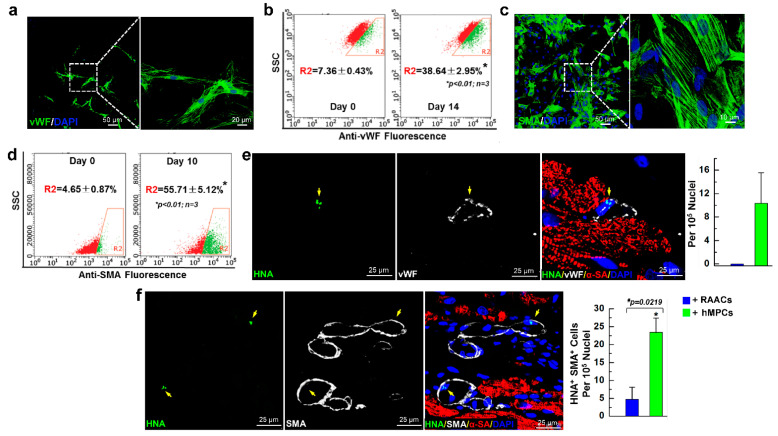
The hMPCs have an ability to differentiate into vascular cells both in vitro and in vivo. (**a**) Representative images of endothelial cells that were differentiated from hMPCs after staining with the von Willebrand factor (vWF, green) endothelial cell maker and DAPI (blue). (**b**) Flow cytometer analysis for vWF positive cells after 14 days of differentiation from hMPCs. (**c**) Representative images of smooth muscle cells that were differentiated from hMPCs after staining with smooth muscle actin (SMA, green) and DAPI (blue). (**d**) Flow cytometer analysis for SMA positive cells after 10 days of differentiation from hMPCs. (**e**) Immunofluorescent staining for the risk area of the infarcted heart with vWF and human nucleic marker (HNA); cells with double-positive staining (yellow arrow) indicate the endothelial cells that were derived from transplanted hMPCs. Quantitative analysis shown in the right panel. The y-axis represents the number of HNA^+^vWF^+^ cells per 10^5^ nuclei. (**f**) Immunofluorescent staining for the risk area of the infarcted heart with SMA and HNA; cells with double-positive staining (yellow arrow) indicate the smooth muscle cells that were derived from transplanted hMPCs. Quantitative analysis shown in the right panel. The y-axis represents the number of HNA^+^SMA^+^ cells per 10^5^ nuclei. Note: 6 mice for each group; 20 images were taken for each section. Data are means ± SEM; * *p* < 0.05, unpaired students’ *t*-test.

Our data suggested that hMPCs are capable of self-renewal, enriching mesenchymal stem cell markers and cardiac precursor markers, as well as expression of marker genes for cardiac lineage cells. Therefore, we evaluated whether hMPCs can differentiate into vascular cells, including both endothelial and smooth muscle cells. First, following the in vitro differentiation protocol of endothelial cells described previously [43], we found that hMPCs are able to differentiate into endothelial cells at day 14, as indicated by the clear structure of endothelial cells with the staining of the Von Willebrand factor (vWF) endothelial cell maker (Figure 6a). The FACS analysis showed that about 38.64% of cells are positive for vWF after 14 days of differentiation (Figure 6b). Second, to evaluate whether hMPCs are able to differentiate into smooth muscle cells, the manufacturer’s instructions were strictly followed for the differentiation of human coronary artery smooth muscle cells using a commercial kit (Sigma). Briefly, hMPCs were cultured in smooth muscle cell differentiation medium for 10 days, and the medium was changed every two days. The results showed that hMPCs can also differentiate into smooth muscle cells, as indicated by the positive staining with smooth muscle actin (SMA) (Figure 6c). The results from the FACS analysis demonstrated that ~55.71% of differentiated cells are positive for SMA staining after 10 days of differentiation (Figure 6d). 

To detect the hMPC-derived cells inside the recipient mouse heart, immunostaining with the antibody against human nucleic antigen (HNA) was performed for heart sections of different groups of animals. Notably, it was observed that transplanted hMPCs have the capacity to differentiate into vascular cells in vivo, as indicated by the immunofluorescent staining of vWF and SMA. As shown in Figure 6e,f (yellow arrow), some hMPC-derived cells are positive for both human nucleic antigen (HNA) and vWF (HNA^+^vWF^+^) (Figure 6e). Additionally, some hMPC-derived cells are positive for both HNA and SMA (HNA^+^SMA^+^) (Figure 6f). Quantitative analysis shows 10.4 ± 5.2 per 10^5^ nuclei are double-positive for HNA and vWF in the risk area of infarcted heart, and 23.4 ± 5.0 per 10^5^ nuclei are double-positive for HNA and SMA in the risk area of the infarcted heart (Figure 6e,f, right panel). Taken together, these data suggest that hMPCs can differentiate into endothelial and smooth muscle cells in both in vitro cell culture conditions and in in vivo animal MI models after intramyocardial cell transplantation.

### 3.7. Administration of hMPCs Enhances Vascular Regeneration in Infarcted Heart

**Figure 7:**
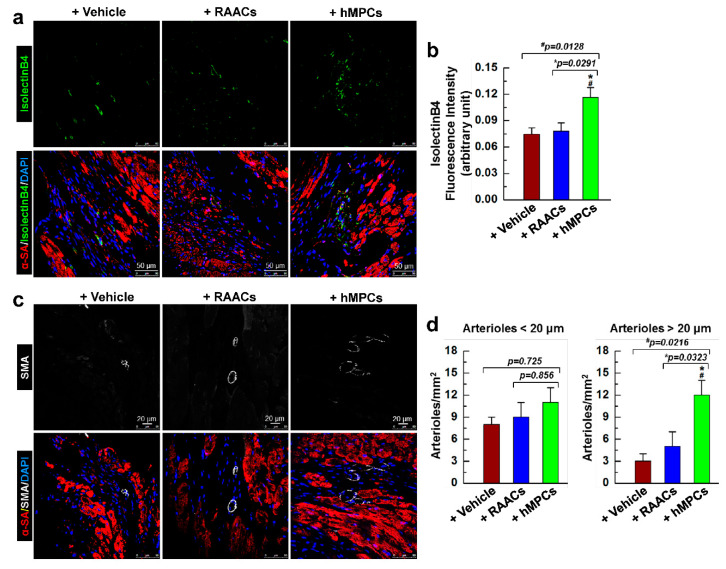
Transplantation of hMPCs enhances the endogenous cardiac vascular regeneration. (**a**) Representative immunohistochemistry images for the staining of endothelial cell maker isolectin B4 (IB4) in the risk area of infarcted mouse hearts. Green color indicates IB4-positive cells, red color indicates α-SA-positive cells, and blue indicates DAPI-stained nuclei. (**b**) Quantitative analysis for the fluorescent intensity of IB4 in different groups of heart section staining. (**c**) Representative confocal images for the staining of SMA in the risk area of infarcted heart sections. Green color indicates SMA-positive cells, red color indicates α-SA-positive cells, and blue color indicates DAPI-stained nuclei. (**d**) Quantitative analysis for SMA positive arterioles smaller or larger than 20 µm in diameter. The y-axis represents the number of arterioles per mm^2^. Note: *n* = 6 mice per group, 20 images/slide. Data are means ± SEM. * *p* < 0.05 versus Vehicle group, ^#^ *p* < 0.05 versus RAAC group, which were calculated by one-way ANOVA followed by Tukey’s multiple comparison test.

To examine whether the infusion of hMPCs is beneficial for endogenous cardiac vascular regeneration in the infarcted hearts, heart sections were stained with isolectin B4 (IB4) conjugated with green fluorescence dye to visualize the vesicular endothelial cells. Interestingly, the results from evaluating confocal images demonstrated that the fluorescent intensity of capillaries was significantly higher in the risk area of the heart for the hMPC group than that in vehicle and RAAC groups, indicating that transplantation of hMPCs has significant beneficial effects on promoting vascular regeneration in infarcted hearts (Figure 7a,b). In addition, heart sections were also stained with the antibody against SMA to label smooth muscle cells in the vessel wall. Compared with vehicle and RAAC groups, the number of arterioles measuring over 20 μm significantly increased in the risk area of hearts from the hMPC group (Figure 7c,d). Thus, the results from the immunostaining of heart sections indicated that transplantation of hMPCs enhances the endogenous vascular regeneration in an immunodeficient mouse MI model.

Indeed, these in vivo results are consistent with the data shown in Figure 3a–d, where hMPCs exhibited higher expression of pro-angiogenesis and cytoprotective cytokines. The in vitro vessel tube formation by endothelial cells was also evaluated using the differentiated hMPCs, and the results showed that differentiated endothelial cells have a capacity to format vessel tubes (Supplementary Figure S6a,b). Hence, hMPCs exhibited the capacity to release more pro-angiogenesis cytokines; therefore, hMPCs contribute to the enhanced new vessel formation or vascular regeneration in the infarcted heart, provide a better microenvironment to supply nutrients and oxygen, and preserve the cardiac function upon I/R-induced myocardial injury.

## 4. Discussion

This study focuses on a subpopulation of adult progenitor cells, hMPCs, which were sorted from human right atrial appendage-derived cells based on the low mitochondrial membrane potential using a similar approach described previously [41]. The results from in vitro studies suggest hMPCs hold the capacity for stemness, exhibit better capacity to proliferate against oxidative-stress-induced cell apoptosis due to increased expression of anti-apoptotic and antioxidant proteins, and release more cytoprotective cytokines compared to the non-sorted RAACs. Our in vivo study with an immunodeficient murine MI model showed that the transplantation of hMPCs into the MI heart alleviates cardiac dysfunction and enhances cardiac vascular regeneration.

It was observed that hMPCs are more like the mesenchymal stem or progenitor cells, as most hMPCs are positive for mesenchymal stem cell markers or cardiac precursor markers, including CD90, CD 44, CD140b, CD172a, and CD172b. Interestingly, compared with RAACs, hMPCs show greater expression of cardiac-specific lineage marker genes and stem cell lineage genes, but there is no significant difference in the expression of the maturity cardiac lineage genes. Notably, our data demonstrated that hMPCs can potentially differentiate into endothelial cells and smooth muscle cells; therefore, we named these progenitor cells hMPCs. When hMPCs were subjected to induced cardiac differentiation, spontaneous beating cells and a clear myocardial structure were rarely observed after four weeks of differentiation. Although we observed ~ 0.16% newly formed BrdU^+^/α-SA^+^ CMs, a very limited number (~0.012%) of these newly formed CMs were derived from transplanted hMPCs following double staining with HNA and α-SA (data not shown), indicating that the newly formed CMs might mainly come from endogenous proliferated or regenerated CMs. Malliaras et al. previously reported that 2.09% BrdU^+^ CMs were observed over the base of GFP positive CMs after 5 weeks of BrdU pulsing for animals that received CDCs [44]. However, these data are not comparable to our results because the methodologies used to calculate BrdU^+^ CMs and the donor cells are different.

Although cell therapy has emerged as a promising new approach to the treatment of heart failure (HF), the process of translating this therapy into clinical study was almost stopped in recent years, mainly due to the debate regarding the contribution of adult cardiac stem or progenitor cells to newly formed cardiomyocytes following myocardial injury. The results from several independent research groups demonstrated that adult cardiac stem cells make a minimal contribution to newly formed cardiomyocytes in Cre-LoxP mice via lineage tracking of the fate of c-kit^+^ CPCs or sca-1^+^ CPCs [36,37,38,39]. In fact, these CPCs are vascular progenitor cells, as the cells can most likely differentiate into the endothelial cells based on the in vivo lineage tracing [36,37,38,39]. However, the other investigators refuted that c-kit haploinsufficiency in the Cre-lox system of c-kit-Cre-KI mice severely affects CSCs’ myogenic potential, and transplantation of wild-type CSCs rescued the defective regenerative cardiac phenotype of c-kit-Cre-KI mice [40]. Most investigators previously focused on cardiac progenitor cells with special cell surface markers that were used for cellular therapy of ischemic heart diseases or heart failure. It has been reported that low mitochondrial membrane potential T cells demonstrate long survival times and enhanced stemness for cell therapy [41]. Other than the present study, there are no reports regarding the use of adult progenitor cells with low mitochondrial membrane potential for cell-based therapy in heart failure.

It has been reported that more than 90% of transplanted cells die within one week and that more than 95% cells die within five weeks [42,45,46]. Notably, compared with RAACs’ lineage, hMPCs exhibit better benefits in terms of cell survival capability, faster cell proliferation, and lower ROS generation, all of which are key factors for enhancing the efficiency of cell-based therapy. Multiple independent groups have shown evidence that adult cardiac progenitor cells mediate tissue repair via the release of paracrine factors into risk tissue, which subsequently benefit a number of restorative processes, including myocardial protection, revascularization, and cardiac remodeling [47,48]. Consistently, our results from the qPCR analysis of the human cytokine library I/II and Western blots of human cytokine antibody library array showed significantly increased expression or greater release of cytoprotective and pro-angiogenetic cytokines in hMPCs than RAACs, including FGF10/16/20/, NTF5, CCL3, CXCL12, CCL11, CXCL6, and others. In addition, conditional medium (CM) collected from hMPCs exhibits the capacity to improve the survival of RAACs, indicating the beneficial effects of the secreted cytoprotective and pro-angiogenetic cytokines. These results also provide mechanistic evidence to support the cardioprotective effect of hMPCs in promoting endogenous cardiac repair processes after transplantation into the infarcted heart. 

Following myocardial infarction, both myocardium and vessels in the heart might be excessively injured. It was reported that new blood vessel formation by delivering angiogenic genes to ischemic tissues appears as a promising alternative therapeutic strategy to treat ischemic heart diseases [49,50,51]. In this study, we observed that hMPCs can secrete more paracrine factors that enhance neovascularization and inhibit cardiac cell apoptosis. The immunohistochemistry results demonstrated significantly increased angiogenesis markers, including IB4 and SMA in the risk area of the infarcted heart with hMPC infusion, indicating greater vascular regeneration. Furthermore, both the qPCR array and antibody array showed that hMPCs exhibit greater gene expression of cytokines and release more pro-angiogenesis cytokines at the protein level, including colony stimulating factor 2 (CSF2), CCL8, bone morphogenetic protein 10 (BMP10), CCL3, CXCL12, FGF20, prolactin (PRL), insulin-like growth factor 1 (IGF-1), angiopoietin-2 (ANGPT2), and erythropoietin (EPO). Interestingly, it was also observed that hMPCs hold the capacity to form vessel tubes in vitro. Taken together, our results clearly support the mechanistic hypothesis that transplantation of hMPCs preserves the cardiac function and morphology by enhancing the angiogenesis in the risk area of infarcted heart.

In the past two decades, researchers have reached significant milestones toward their goal of using stem or progenitor cells as a regenerative treatment to the bedside [52]. Herein, we provide one more piece of evidence in support of adult hMPCs as potential and efficient cell sources for cell-based therapy for ischemic heart disease. However, there are several limitations in this study. First, hMPCs are a mixed cell population, in which isolation was based on the low mitochondrial membrane potential. This might lead to the loss of other useful cell populations with higher mitochondrial membrane potential in the human heart. Second, although some of the hMPCs express the mature cardiac marker protein after cardiac differentiation, spontaneous beating cells were never observed after long-term cardiac differentiation, and it was observed that newly formed cardiomyocytes derived from hMPCs were rarely found in the risk area of the infarcted heart. Therefore, the cardioprotective effects of hMPCs might not directly contribute to the myocardium regeneration other than promoting angiogenesis and enhancing the endogenous cardiac regeneration. Third, there are other potential factors that might be associated with the beneficial effect of hMPCs. For example, the expression of specific non-coding RNAs and release of exosomes and other extracellular vesicles cannot be ruled out and need to be further investigated. Finally, further studies with large animal models may need to be conducted to validate the results presented here.

## 5. Conclusions

In summary, this study investigated a new subpopulation of adult progenitor cells, hMPCs, which preserve heart function upon transplantation of these cells into the infarcted heart. The mechanistic action of hMPCs might be associated with the secretion of angiogenesis paracrine factors, thereby enhancing cardiac vascular regeneration. These results demonstrate that hMPCs might serve as potential and effective donor cells for the cell therapy of ischemic heart disease. Furthermore, our data show that adult human progenitor cell-based therapy is an alternative and promising strategy to protect against cardiac dysfunction in patients with ischemic heart diseases.

## Data Availability

Not applicable.

## Supplementary Materials

The following supporting information can be downloaded at: https://www.mdpi.com/article/10.3390/cells11101713/s1: Figure S1: Flow cytometric analysis of cell surface markers in hMPCs. Figure S2: Flow cytometric analysis of cell surface markers in hMPCs. Figure S3: Heart rates for animals during the echocardiographic measurements. Figure S4: Transplantation of hMPCs into female infarcted mouse heart improves cardiac function and structure. Figure S5: Transplantation of hMPCs reduces the cardiac fibrosis in infarcted mouse heart. Figure S6: Transplantation of hMPCs reduces the cardiac fibrosis in infarcted mouse heart. Table S1: Clinical profile of patients used for hMPC isolation. Table S2: List of genes in the human cytokine primer library I. Table S3: List of genes in the human cytokine primer library II. Table S4: Human cytokine antibody library array (Cat. NO. AAH-CYT-6-2). Table S5: Primer sequence for RT-PCR. Table S6: Key resource table. Detailed Supplementary Methods. References [10,41,42,43] are cited in Supplementary Materials.
