# Supplementary material for: Mitochondrial Membrane Potential Identifies a Subpopulation of Mesenchymal Progenitor Cells to Promote Angiogenesis and Myocardial Repair
Source: Cells. 2022 May 22;11(10):1713. doi: 10.3390/cells11101713 (PMC9139404; doi:10.3390/cells11101713)
Supplement: Supplementary file 1 [file cells-11-01713-s001.zip › Supplementary Figures and Tables.pdf]

**Supplementary Figures and Tables**

# Mitochondrial Membrane Potential Identifies a Subpopulation of Mesenchymal Progenitor Cells to Promote Angiogenesis and Myocardial Repair

**Xiuchun Li [1,2,†], Xiaoliang Wang [1,2,†], Pan He [2], Edward Bennett [3], Erin Haggard [1], Jianjie Ma [1] and Chuanxi Cai [1,2,*]**

[1] Department of Surgery, Davis Heart and Lung Research Institute, The Ohio State University Wexner Medical Center, Columbus, OH 43210, USA; lxchosu@hotmail.com (X.L.); xiaoliang.wang@osumc.edu (X.W.); erin.haggard@osumc.edu (E.H.); jianjie.ma@osumc.edu (J.M.)
[2] Department of Molecular and Cellular Physiology, Albany Medical College, Albany, NY 12208, USA; hpan2015@hotmail.com
[3] Division of Cardiothoracic Surgery, Albany Medical Center, Albany, NY 12208, USA; bennette@mail.amc.edu
* Correspondence: chuanxi.cai@osumc.edu; Tel.: +1-614-292-2213
† These authors contributed equally to this work.

**Supplementary Figures**

**Supplementary Figure S1. Flow cytometric analysis of cell surface markers in hMPCs.** Flow cytometer analysis of cell surface markers in hMPCs, including mesenchymal stem cell markers (CD90, CD44 and CD140b) and cardiac precursor cell markers (CD117, CD172a and CD172b). Green cell populations are positive cells for the individual cell surface marker as indicated in the images. Data are mean ± SEM, n=3.

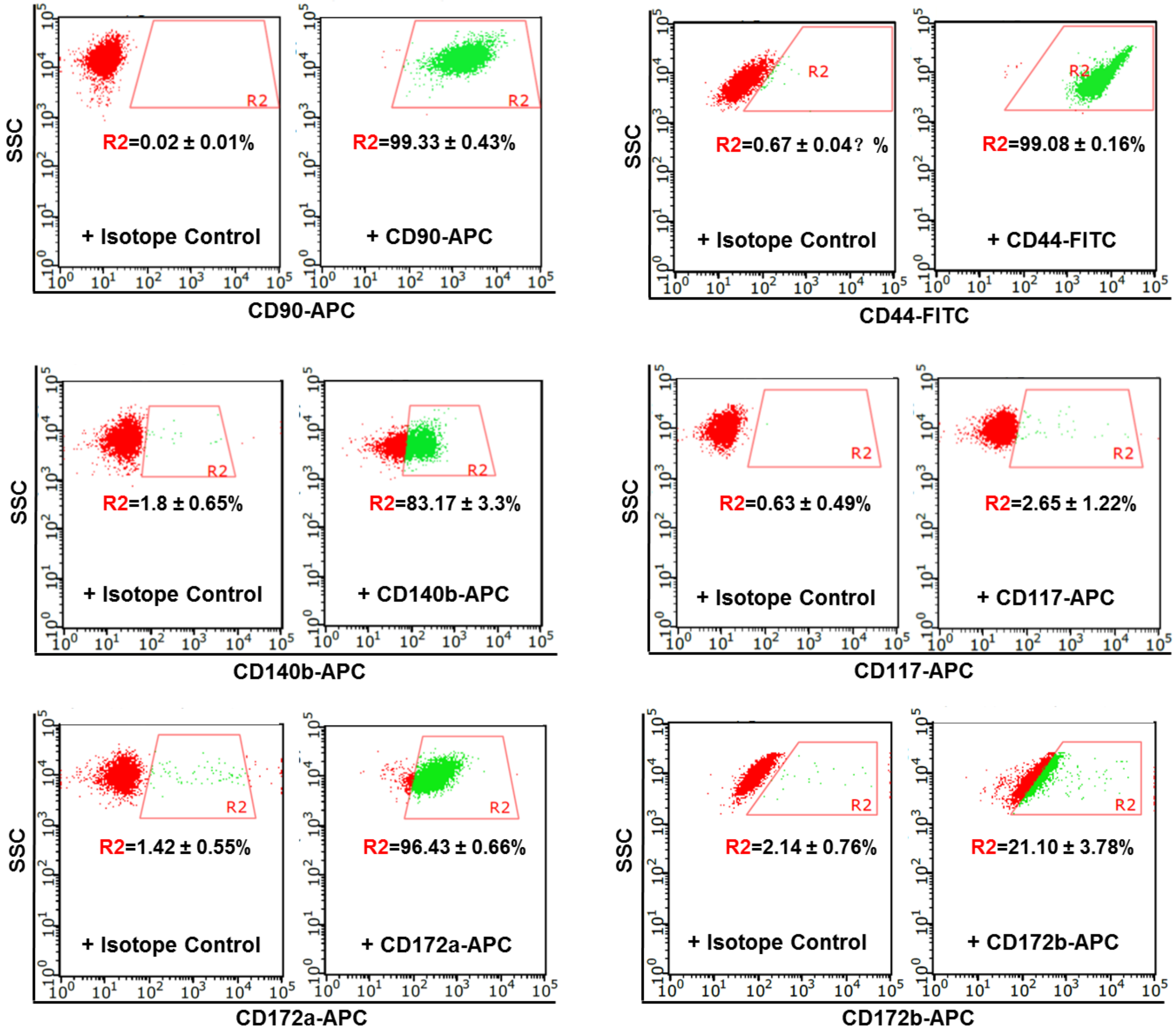

**Supplementary Figure S2. Flow cytometric analysis of cell surface markers in hMPCs.** Flow cytometer analysis of cell surface markers in hMPCs, including hematopoietic stem cell markers (CD31, CD45 and CD133) and angiogenic cell markers (CD146, CD184 and CD309). Green cell populations are positive cells for the individual cell surface marker as indicated in the images. Data are mean ± SEM, n=3.

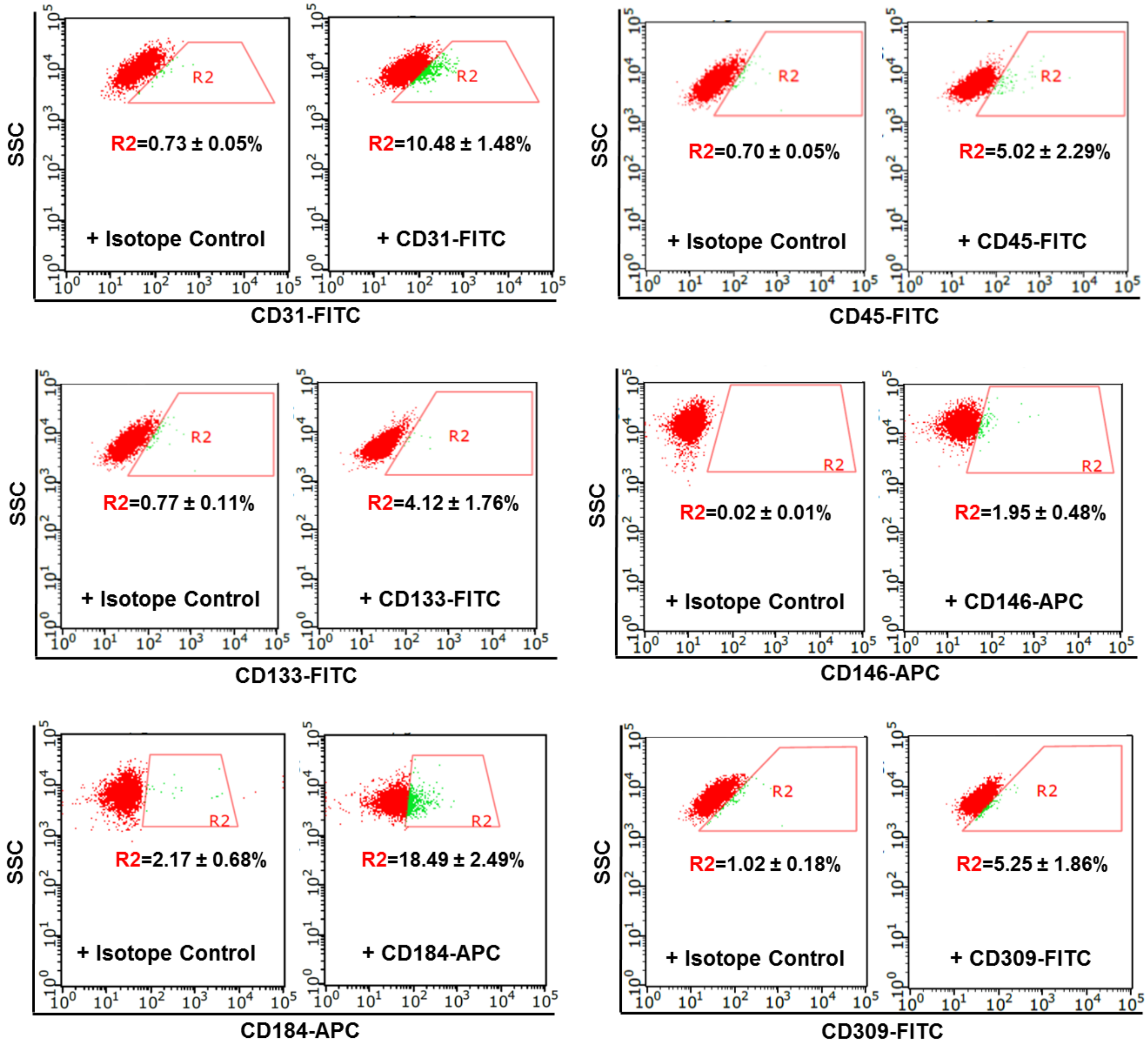

**Supplementary Figure S3. Heart rate for animals during the echocardiographic measurement. a**. Schematic animal model for the myocardial infarction induced by ischemia/reperfusion, and the injection sites for donor cell transplantation. **b-c**. Quantitative analysis of heart rate that were recorded during conducting the echocardiographic measurement for both male (**b**) and female (**c**) mice. "BSL" stands for "baseline".

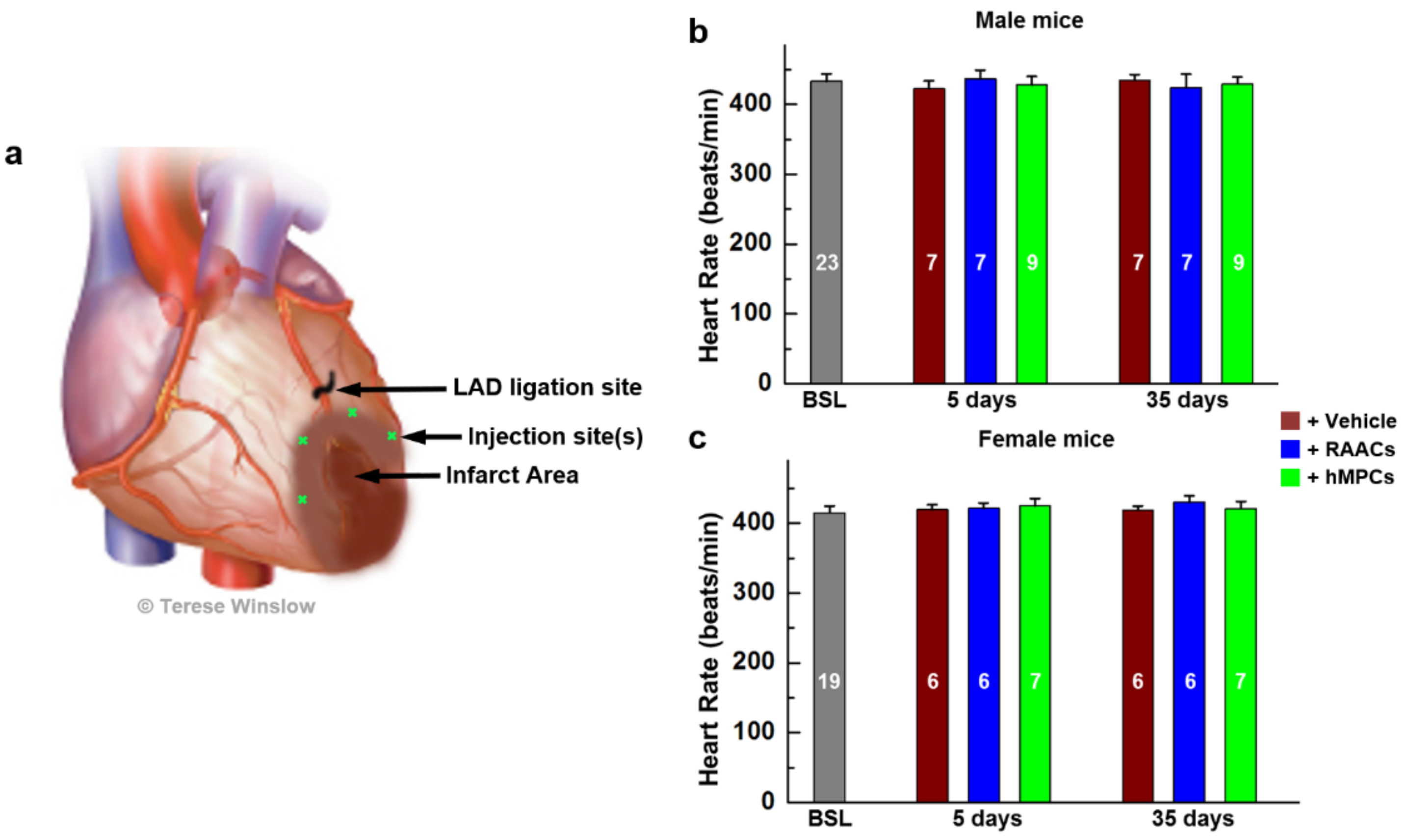

**Supplementary Figure S4. Transplantation of hMPCs into female mouse infarcted heart improves cardiac function and structure. a**. Echocardiographic parameters for female mice, including ejection fraction (EF), left ventricle anterior wall thickness (LVAWT), left ventricle posterior wall thickness (LVPWT), LV volume-dystonic, LV volume-systolic, and LV mass among the groups of vehicles (n=6), RAACs (n=6) and hMPCs (n7) at 5 days and 35 days after cell transplantation. "BSL" stand for "baseline". **b**. Hemodynamic parameters for the female mice among three groups, including EF, stoke volume, $dP/dt_{max}$, $dP/dt_{min}$ and cardiac output at 39 days. Data are mean ± SEM. * $p < 0.05$ versus Vehicle group, $^{\#} p < 0.05$ versus RAAC group, which was calculated by the one-way ANOVA followed by Tukey's multiple comparison test.

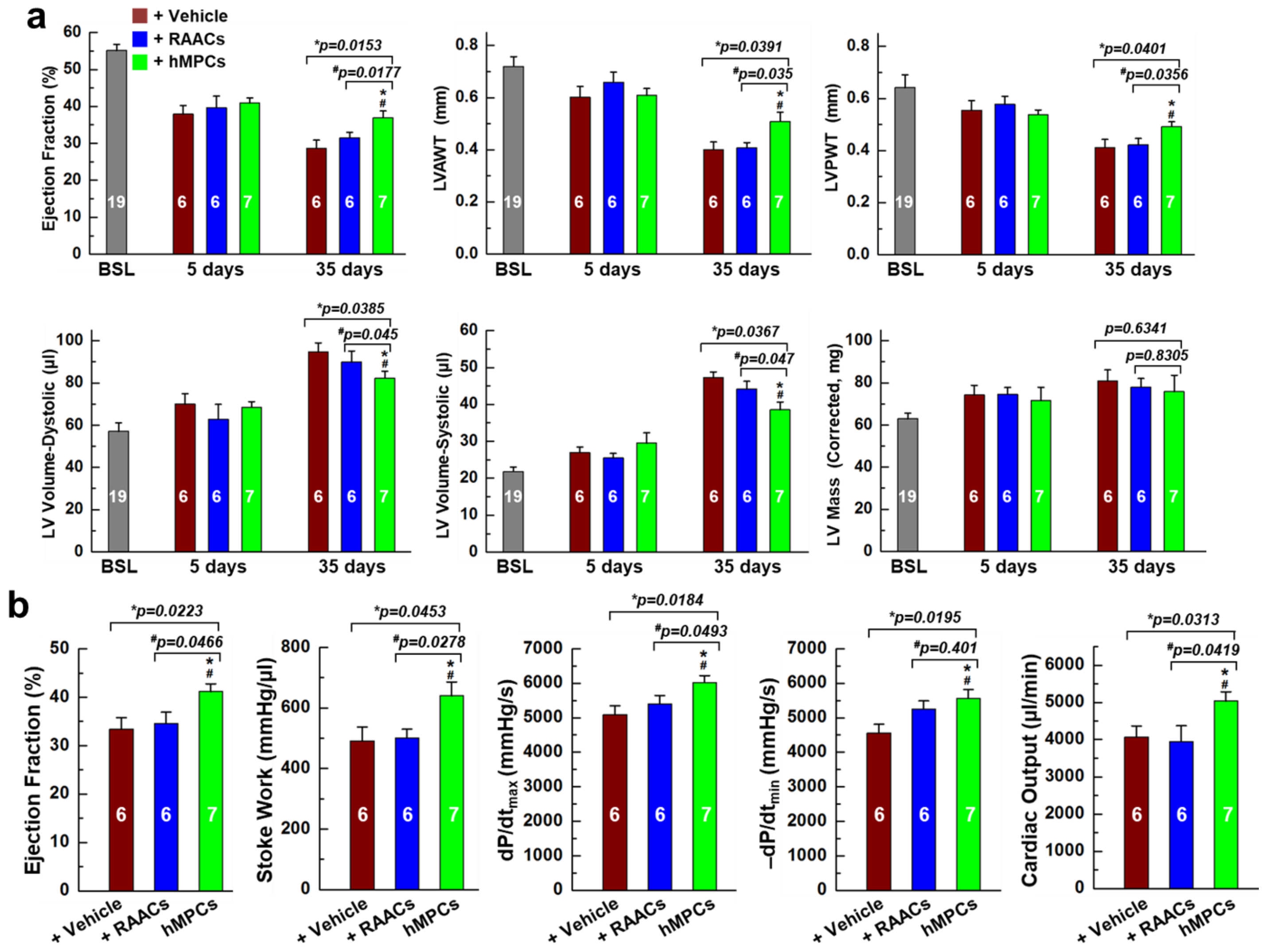

**Supplementary Figure S5 Transplantation of hMPCs reduces the cardiac fibrosis in infarcted mouse heart. a**. Representative confocal images for cardiac fibrosis followed with the Wheat Germ Agglutinin (WGA) staining. Bright green area indicated the fibrosis area in heart. **b**. Quantitative analysis for the percentage of cardiac fibrosis. The y-axis represents the percentage of the fibrotic area over the whole heart area. Data are mean ± SEM. * $p < 0.05$ versus Vehicle group, # $p < 0.05$ versus RAAC group , which was calculated by the one-way ANOVA followed by Tukey's multiple comparison test.

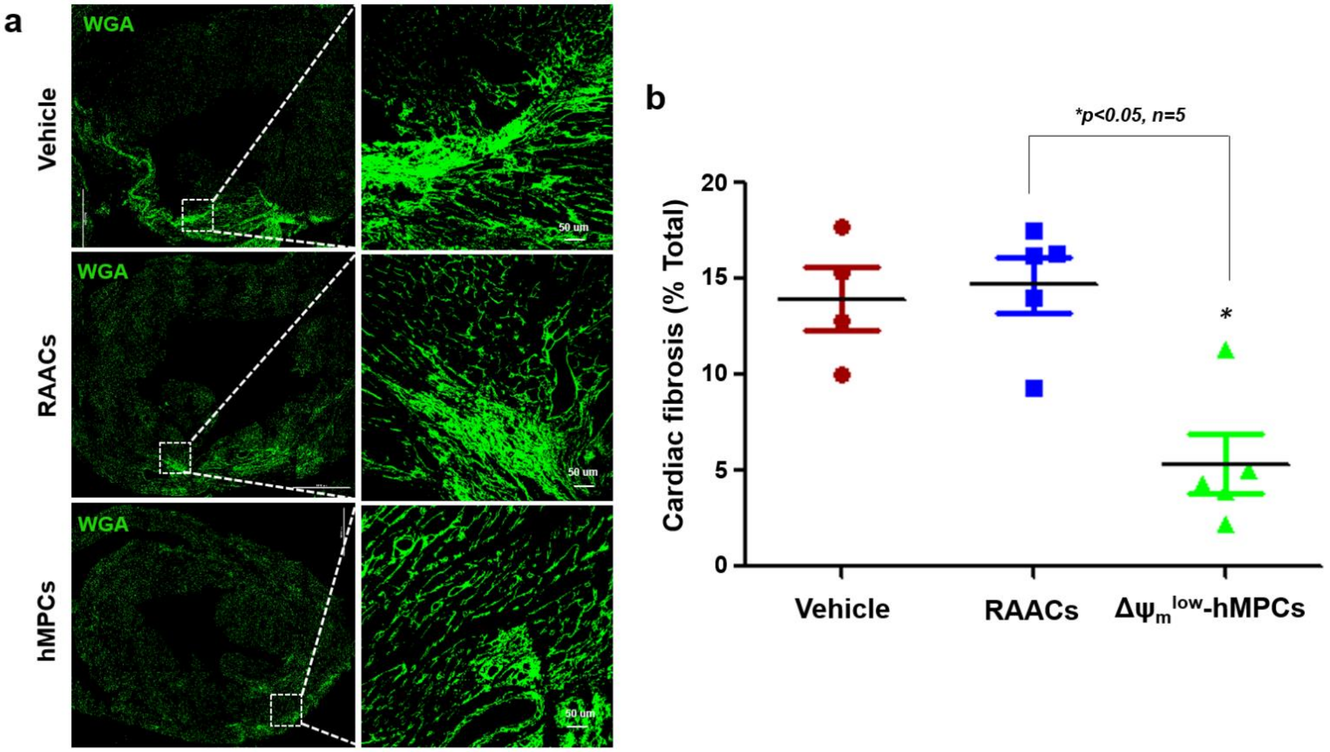

**Supplementary Figure S6 hMPCs has the capacity to form vessel tubes in vitro. a**. Representative images of endothelial cells that were differentiated from hMPCs following with the staining with vWF antibody. Green color indicates vWF positive cells, and blue color indicates the DAPI stained nuclei. **b**. Representative image for vessel tubes formation derived from hMPCs *in vitro*.

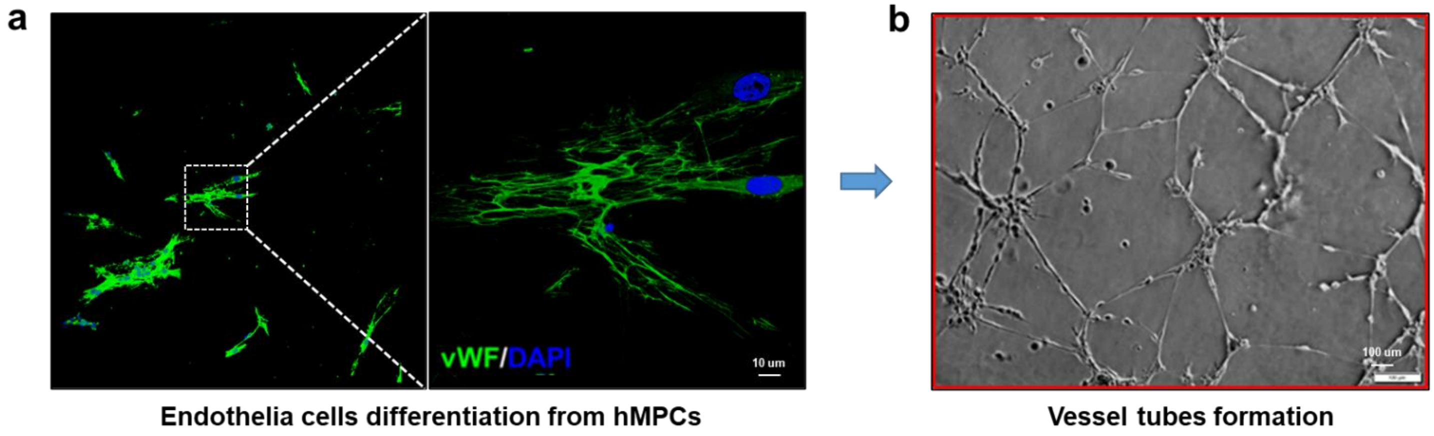

## Supplementary Tables

**Supplemental Table S1.** Clinical Profile of Patients used for hMPCs Isolation.

| Patient ID | Age (Yrs) | Sex | EF% | Cardiac Index | Diabetes | Hyper-lipidemia | Smoker | Infarct | Ischemia | ACE Inhibitor | β-Blocker | Anti-coagulant | NYHA |
|---|---|---|---|---|---|---|---|---|---|---|---|---|---|
| AMC119 | 74 | Male | 50% | - | x | - | √ | √ | √ | √ | √ | √ | III |
| AMC123 | 74 | Male | 57% | 1.68 | x | - | x | √ | √ | - | - | √ | III |
| AMC131 | 42 | Male | 48% | 1.52 | x | √ | √ | √ | √ | √ | √ | √ | IV |
| AMC143 | 70 | Male | 55% | - | x | - | - | √ | √ | √ | √ | - | III |
| AMC167 | 69 | Male | 60% | 1.57 | x | √ | x | √ | √ | √ | √ | √ | IV |
| AMC175 | 74 | Male | 55% | 1.65 | x | √ | √ | √ | √ | - | √ | - | III |

EF indicates ejection fraction; NYHA, New York Heart Association; Patient information is ( √ ) positive; (x) negative; (-) unavailable.

**Supplemental Table S2**. List of genes in the human cytokine primer library I.

| Well | Symbol | Name | Well | Symbol | Name |
|---|---|---|---|---|---|
| A1 | IL2 | Interleukin 2 | E1 | CSF1 | Colony stimulating factor 1 (macrophage) |
| A2 | IL7 | Interleukin 7 | E2 | FLT3LG | Fms-related tyrosine kinase 3 ligand |
| A3 | IL9 | Interleukin 9 | E3 | FGF1 | Fibroblast growth factor 1 (acidic) |
| A4 | IL15 | Interleukin 15 | E4 | FGF2 | Fibroblast growth factor 2 (basic) |
| A5 | IL21 | Interleukin 21 | E5 | FGF4 | Fibroblast growth factor 4 |
| A6 | TSLP | Thymic stromal lymphopoietin | E6 | FGF5 | Fibroblast growth factor 5 |
| A7 | IL3 | Interleukin 3 (colony-stimulating factor, mu | E7 | FGF6 | Fibroblast growth factor 6 |
| A8 | IL5 | Interleukin 5 (colony-stimulating factor, eo: | E8 | FGF7 | Galactokinase 2 |
| A9 | CSF2 | Colony stimulating factor 2 (granulocyte- m | E9 | FGF8 | Fibroblast growth factor 8 (androgen-induced) |
| A10 | IL6 | Interleukin 6 (interferon, beta 2) | E10 | FGF9 | Fibroblast growth factor 9 (glia-activating factor) |
| A11 | IL11 | Interleukin 11 | E11 | FGF10 | Fibroblast growth factor 10 |
| A12 | IL12A | Interleukin 12A | E12 | FGF11 | Fibroblast growth factor 11 |
| B1 | IL12B | Interleukin 12B | F1 | FGF12 | Fibroblast growth factor 12 |
| B2 | IL23A | Interleukin 23, alpha subunit p19 | F2 | FGF13 | Fibroblast growth factor 13 |
| B3 | LIF | Leukemia inhibitory factor | F3 | FGF14 | Fibroblast growth factor 14 |
| B4 | ZFP91 | Zinc finger protein 91 homolog (mouse) | F4 | FGF16 | Fibroblast growth factor 16 |
| B5 | CLC | Cardiotrophin-like cytokine factor 1 | F5 | FGF18 | Fibroblast growth factor 18 |
| B6 | CTF1 | Cardiotrophin 1 | F6 | FGF19 | Fibroblast growth factor 19 |
| B7 | CSF3 | Colony stimulating factor 3 (granulocyte) | F7 | FGF20 | Fibroblast growth factor 20 |
| B8 | LEP | Leptin (obesity homolog, mouse) | F8 | FGF23 | Fibroblast growth factor 23 |
| B9 | EPO | Erythropoietin | F9 | NRG1 | Neuregulin 1 |
| B10 | GH1 | Growth hormone 1 | F10 | NRG2 | Neuregulin 2 |
| B11 | GH2 | Growth hormone 2 | F11 | NRG3 | Neuregulin 3 |
| B12 | PRL | Prolactin | F12 | NRG4 | Neuregulin 4 |
| C1 | TPO | Thyroid peroxidase | G1 | IGF1 | Insulin-like growth factor 1 (somatomedin C) |
| C2 | IFNA1 | Interferon, alpha 1 | G2 | HGF | Hepatocyte growth factor |
| C3 | IFNB1 | Interferon, beta 1, fibroblast | G3 | EGF | Epidermal growth factor (beta-urogastrone) |
| C4 | IFNW1 | Interferon, omega 1 | G4 | KITLG | KIT ligand |
| C5 | IFNK | Interferon, kappa | G5 | EFNA1 | Ephrin-A1 |
| C6 | IFNE1 | Interferon epsilon 1 | G6 | EFNA2 | Ephrin-A2 |
| C7 | IL28A | Interleukin 28A (interferon, lambda 2) | G7 | EFNA3 | Ephrin-A3 |
| C8 | IL29 | Interleukin 29 (interferon, lambda 1) | G8 | EFNA4 | Ephrin-A4 |
| C9 | IFNG | Interferon, gamma | G9 | EFNA5 | Ephrin-A5 |
| C10 | IL10 | Interleukin 10 | G10 | EFNB1 | Ephrin-B1 |
| C11 | IL20 | Interleukin 20 | G11 | EFNB2 | Ephrin-B2 |
| C12 | IL24 | Interleukin 24 | G12 | EFNB3 | Ephrin-B3 |
| D1 | IL22 | Interleukin 22 | H1 | GAS6 | Growth arrest-specific 6 |
| D2 | IL26 | Interleukin 26 | H2 | ANGPT1 | Angiopoietin 1 |
| D3 | VEGFA | Vascular endothelial growth factor | H3 | ANGPT2 | Angiopoietin 2 |
| D4 | VEGFB | Vascular endothelial growth factor B | H4 | ANGPT4 | Angiopoietin 4 |
| D5 | VEGFC | Vascular endothelial growth factor C | H5 | ACTB | Actin, beta |
| D6 | PDGFC | Platelet derived growth factor C | H6 | B2M | Beta-2-microglobulin |
| D7 | PDGFD | DNA-damage inducible protein 1 | H7 | GAPD | Glyceraldehyde-3-phosphate dehydrogenase |
| D8 | NGFB | Nerve growth factor, beta polypeptide | H8 | GUSB | Glucuronidase, beta |
| D9 | NTF3 | Neurotrophin 3 | H9 | HPRT1 | Hypoxanthine phosphoribosyltransferase 1 |
| D10 | NTF5 | Neurotrophin 5 (neurotrophin 4/5) | H10 | PGK | Phosphoglycerate kinase 1 |
| D11 | BDNF | Brain-derived neurotrophic factor | H11 | PPIA | Peptidylprolyl isomerase A |
| D12 | GDNF | Glial cell derived neurotrophic factor | H12 | RPL13A | Ribosomal protein L13a |

**Supplemental Table S3**. List of genes in the human cytokine primer library II.

| Well | Symbol | Name | Well | Symbol | Name |
|---|---|---|---|---|---|
| A1 | TNF | Tumor necrosis factor (TNF superfamily, member 2) | E1 | INHBA | Inhibin, beta A |
| A2 | LTA | Lymphotoxin alpha (TNF superfamily, member 1) | E2 | INHBB | Inhibin, beta B |
| A3 | LTB | Lymphotoxin beta (TNF superfamily, member 3) | E3 | INHBC | Inhibin, beta C |
| A4 | TNFSF4 | Tumor necrosis factor (ligand) superfamily, member 4 | E4 | INHBE | Inhibin, beta E |
| A5 | CD40LG | CD40 ligand (TNF superfamily) | E5 | TGFB1 | Transforming growth factor, beta 1 |
| A6 | FASLG | Fas ligand (TNF superfamily, member 6) | E6 | TGFB2 | Transforming growth factor, beta 2 |
| A7 | TNFSF7 | CD70 molecule | E7 | TGFB3 | Transforming growth factor, beta 3 |
| A8 | TNFSF8 | Tumor necrosis factor (ligand) superfamily, member 8 | E8 | AMH | Anti-Mullerian hormone |
| A9 | TNFSF9 | Tumor necrosis factor (ligand) superfamily, member 9 | E9 | INHA | Inhibin, alpha |
| A10 | TNFSF10 | Tumor necrosis factor (ligand) superfamily, member 10 | E10 | BMP10 | Bone morphogenetic protein 10 |
| A11 | TNFSF11 | Tumor necrosis factor (ligand) superfamily, member 11 | E11 | LEFTY1 | Left-right determination factor 1 |
| A12 | TNFSF13 | Tumor necrosis factor (ligand) superfamily, member 13 | E12 | LEFTY2 | Left-right determination factor 2 |
| B1 | TNFSF13B | Tumor necrosis factor (ligand) superfamily, member 13b | F1 | CCL2 | Chemokine (C-C motif) ligand 2 |
| B2 | TNFSF14 | Tumor necrosis factor (ligand) superfamily, member 14 | F2 | CCL3 | Chemokine (C-C motif) ligand 3 |
| B3 | TNFSF15 | Tumor necrosis factor (ligand) superfamily, member 15 | F3 | CCL4 | Chemokine (C-C motif) ligand 4 |
| B4 | TNFSF18 | Tumor necrosis factor (ligand) superfamily, member 18 | F4 | CCL5 | Chemokine (C-C motif) ligand 5 |
| B5 | EDA | Ectodysplasin A | F5 | CCL7 | Chemokine (C-C motif) ligand 7 |
| B6 | IL1A | Interleukin 1, alpha | F6 | CCL8 | Chemokine (C-C motif) ligand 8 |
| B7 | IL1B | Interleukin 1, beta | F7 | CCL11 | Chemokine (C-C motif) ligand 11 |
| B8 | IL1RN | Interleukin 1 receptor antagonist | F8 | CCL13 | Chemokine (C-C motif) ligand 13 |
| B9 | IL18 | Interleukin 18 (interferon-gamma-inducing factor) | F9 | CCL15 | Chemokine (C-C motif) ligand 14 |
| B10 | IL1F5 | Interleukin 1 family, member 5 (delta) | F10 | CCL16 | Chemokine (C-C motif) ligand 16 |
| B11 | IL1F7 | Interleukin 1 family, member 7 (zeta) | F11 | CCL17 | Chemokine (C-C motif) ligand 17 |
| B12 | IL1F8 | Interleukin 1 family, member 8 (eta) | F12 | CCL18 | Chemokine (C-C motif) ligand 18 |
| C1 | IL1F9 | Interleukin 1 family, member 9 | G1 | CCL19 | Chemokine (C-C motif) ligand 19 |
| C2 | IL1F10 | Interleukin 1 family, member 10 (theta) | G2 | CCL20 | Chemokine (C-C motif) ligand 20 |
| C3 | IL17 | Interleukin 17 | G3 | CCL21 | Chemokine (C-C motif) ligand 21 |
| C4 | IL17B | Interleukin 17B | G4 | CCL22 | Chemokine (C-C motif) ligand 22 |
| C5 | IL17C | Interleukin 17C | G5 | CCL23 | Chemokine (C-C motif) ligand 23 |
| C6 | IL17D | Interleukin 17D | G6 | CCL24 | Chemokine (C-C motif) ligand 24 |
| C7 | IL17E | Interleukin 17E | G7 | CCL25 | Chemokine (C-C motif) ligand 25 |
| C8 | IL17F | Interleukin 17F | G8 | CCL26 | Chemokine (C-C motif) ligand 26 |
| C9 | BMP2 | Bone morphogenetic protein 2 | G9 | CCL27 | Chemokine (C-C motif) ligand 27 |
| C10 | BMP4 | Bone morphogenetic protein 4 | G10 | CCL28 | Chemokine (C-C motif) ligand 28 |
| C11 | BMP6 | Bone morphogenetic protein 6 | G11 | CXCL1 | Chemokine (C-X-C motif) ligand 1 |
| C12 | BMP7 | Bone morphogenetic protein 7 | G12 | CXCL2 | Chemokine (C-X-C motif) ligand 2 |
| D1 | BMP8A | Bone morphogenetic protein 8a | H1 | CXCL3 | Chemokine (C-X-C motif) ligand 3 |
| D2 | BMP8B | Bone morphogenetic protein 8b | H2 | CXCL5 | Chemokine (C-X-C motif) ligand 5 |
| D3 | GDF5 | Growth differentiation factor 5 | H3 | CXCL12 | Chemokine (C-X-C motif) ligand 12 |
| D4 | GDF7 | Growth differentiation factor 7 | H4 | CXCL13 | Chemokine (C-X-C motif) ligand 13 |
| D5 | LASS1 | LAG1 longevity assurance homolog 1 | H5 | ACTB | Actin, beta |
| D6 | GDF3 | Growth differentiation factor 3 | H6 | B2M | Beta-2-microglobulin |
| D7 | BMP3 | Bone morphogenetic protein 3 | H7 | GAPD | Glyceraldehyde-3-phosphate dehydr |
| D8 | GDF10 | Growth differentiation factor 10 | H8 | GUSB | Glucuronidase, beta |
| D9 | GDF8 | Growth differentiation factor 8 | H9 | HPRT1 | Hypoxanthine phosphoribosyltransfe |
| D10 | GDF11 | Growth differentiation factor 11 | H10 | PGK | Phosphoglycerate kinase 1 |
| D11 | GDF9 | Growth differentiation factor 9 | H11 | PPIA | Peptidylprolyl isomerase A |
| D12 | BMP15 | Bone morphogenetic protein 15 | H12 | RPL13A | Ribosomal protein L13a |

**Supplemental Table S4**. Human Cytokine antibody Library array (Cat. NO. AAH-CYT-6-2)

Each antibody is spotted in duplicate vertically

| | A | B | C | D | E | F | G | H | I | J | K | L | M | N |
|---|---|---|---|---|---|---|---|---|---|---|---|---|---|---|
| 1<br>2 | POS | POS | NEG | NEG | BLANK | Angiogenin | BDNF | BLC (CXCL13) | BMP-4 | BMP-6 | Ck beta 8-1 (CCL23) | CNTF | EGF | Eotaxin-1 (CCL11) |
| 3<br>4 | Eotaxin-2 (CCL24) | Eotxin-2 (CCL26) | FGF-6 | FGF-7 (KGF) | Flt-3 Ligand | Fractalkine (CX3CL1) | GCP-2 (CXCL6) | GDNF | GM-CSF | I-309 (CCL1) | IFN-gamma | IGFBP-1 | IGFBP-2 | IGFBP-4 |
| 5<br>6 | IGF-1 | IL-10 | IL-13 | IL-15 | IL-16 | IL-1 alpha (IL-1 F1) | IL-1 beta (IL-1 F2) | IL-1 ra (IL-1 F3) | IL-2 | IL-3 | IL-4 | IL-5 | IL-6 | IL-7 |
| 7<br>8 | Leptin | LIGHT (TNFSF14) | MCP-1 (CCL2) | MCP-2 (CCL8) | MCP-3 (MARC/CCL7) | MCP-4 (CCL13) | M-CSF | MDC (CCL22) | MIG (CXCL9) | MIP-1 delta (CCL15) | MIP-3 alpha (CCL20) | NAP-2 (CXCL7) | NT-3 | PARC (CCL18) |
| 9<br>10 | PDGF-BB | RANTES (CCL5) | SCF | SDF-1 alpha | TARC (CCL17) | TGF beta 1 | TGF beta 3 | TNF alpha | TNF beta | BLANK | BLANK | BLANK | BLANK | POS |

**Supplementary Table S5**. Primer sequence for RT-PCR

| Primer Name | Sequences | Primer Name | Sequences |
|---|---|---|---|
| Myc F | AGCAGAGGAGCAAAAGCTCATT | Nkx2.5 F | ACCTCAACAGCTCCCTGACTCT |
| Myc R | CCAAAGTCCAATTTGAGGCAGT | Nkx2.5 R | ATAATCGCCGCCACAAACTCTCC |
| Sox2 F | TTACCTCTTCCTCCCACTCCAG | OCT3/4 F | GCTTCAGACTTCGCCTCCTCACC |
| Sox2 R | GGGTTTTCTCCATGCTGTTTCT | OCT3/4 R | GCCATCCCTCCGCAGAACTCG |
| Lin28 F | CAAAAGGAAAGAGCATGCAGAA | RYR2 F | GTCTTCCTCAGACACCACCG |
| Lin28 R | ATGATCTAGACCTCCAGAGTTGTAGC | RYR2 R | TGATGAGGGAGCCTGGATGA |
| DPPA3 F | AACCCTGGTCCCAGTCTTCA | ESRRG F | TCAGAACGGACTTGACTCGC |
| DPPA3 Rr | GCAGCATCTGAAATGTCCAC | ESRRG R | ACACAGTCTCTTGGGCATCG |
| p16 F | GACATCCCCGATTGAAAGAA | MESP1 F | CCTCCTGCTTGCCTCAAAGT |
| p16 R | TTTACGGTAGTGGGGGAAGG | MESP1 R | CGAAGTGGTTCCTTGGCAGA |
| MYH6 F | CAAGTTGGAAGACGAGTGCT | MYOCD F | AAAAAGCCCAAGGACCCCAA |
| MYH6 R | ATGGGCCTCTTGTAGAGCTT | MYOCD R | AGTCCATAGGTGGAGGGGAC |
| cTNT F | TTCACCAAAGATCTGCTCCTCGCT | GATA6 F | CTTCCCCCACAACACAACCT |
| cTNT R | TTATTACTGGTGTGGAGTGGGTGTGG | GATA6 R | CTGTTCTCGGGATTGGTGCT |
| GAPDH F | GGAGCGAGATCCCTCCAAAAT | NANOG F | AGAAGGCCTCAGCACCTAC |
| GAPDH R | GGCTGTTGTCATACTTCTCATGG | NANOG R | GGCCTGATTGTTCCAGGAT |
| GATA4 F | ACCTGGGACTTGGAGGATAGCAAA | p21 F | ATTAGCAGCGGAACAAGGAGTCAGACAT |
| GATA4 R | TCCCATCAGCGTGTAAAGGCATCT | p21 R | CTGTGAAAGACACAGAACAGTACAGGGT |
| Mef2C F | AGATACCCACAACACACCAGGCGCC | p27 F | TAACCCGGGACTTGGAGAAG |
| Mef2C R | ATCCTTCAGAGAGTCGCATGCGCTT | p27 R | GCTTCTTGGGCGTCTGCTC |
| Hand2 F | CATCGCCTACCTCATGGACC | | |
| Hand2 R | TCTTGTCGTTGCTGCTCACT | | |

**Supplementary Table S6**. Key resource table

| REAGENT or RESOURCE | SOURCE | IDENTIFIER |
|---|---|---|
| **Antibodies** | | |
| Anti-CD90-APC (clone DG53) | Miltenyi Biotec | Cat#130-097-935 |
| Anti-CD44-FITC (clone DG53) | Miltenyi Biotec | Cat#130-113-896 |
| Anti-CD140b-APC (clone REA363) | Miltenyi Biotec | Cat#130-105-322 |
| Anti-CD117-APC (clone A3C6E2) | Miltenyi Biotec | Cat#130-091-733 |
| Anti-CD172a-APC (clone REA144) | Miltenyi Biotec | Cat#130-099-785 |
| Anti-CD172a-APC (clone B4B6) | Miltenyi Biotec | Cat#130-105-484 |
| Anti-CD31-FITC (clone REA730) | Miltenyi Biotec | Cat#130-110-668 |
| Anti-CD45-FITC (clone 5B1) | Miltenyi Biotec | Cat#130-098-043 |
| Anti-CD133/2 VioBright FITC (clone293C3) | Miltenyi Biotec | Cat#130-104-322 |
| Anti-CD309-VioBright FITC (clone ES8-20E6) | Miltenyi Biotec | Cat#130-105-303 |
| Anti-CD146-APC (clone 541-10B2) | Miltenyi Biotec | Cat#130-097-942 |
| Anti-CD184-APC (clone REA649) | Miltenyi Biotec | Cat#130-109-886 |
| Mouse monoclonal Anti-Cardiac Troponin T-FITC (clone 1C11) | Abcam | Cat#ab105439 |
| Mouse monoclonal anti-Nkx2.5 (clone 2E1) | Abcam | Cat#ab91196 |
| Mouse Monoclonal Anti- HMOX1 (clone HO-1-1) | Thermo Fisher | Cat#MA1-112 |
| Mouse monoclonal Anti-Cardiac Troponin T-FITC (clone 1C11) | Abcam | Cat#ab8295 |
| Mouse Monoclonal Anti-a-SARCOMERIC ACTIN (clone 5c5) | Sigma | Cat#A2172 |
| Mouse Monoclonal Anti-a Smooth Muscle Actin (clone 1A4) | Sigma | Cat#A2547 |
| Polyclonal Rabbit Anti-von Willebrand Factor | Sigma | Cat#F3520 |
| Goat anti-Mouse IgG (H+L) Cross-Adsorbed Secondary Antibody, Alexa Fluor 488 conjugate | Thermo Fisher | Cat#A-11001 |
| Goat anti-Mouse IgM Heavy Chain Secondary Antibody, Alexa Fluor® 647 conjugate | Thermo Fisher | Cat#A-21238 |
| BrdU (Bu20a) Mouse mAb | Cell signaling | Cat#5292s |
| mouse monoclonal IgG Anti-GAPDH (0411) | Santa Cruz | Cat#sc-47724 |
| Polyclonal Rabbit Anti-$\alpha$-Tubulin | Cell signaling | Cat#2144s |
| Wheat Germ Agglutinin (Alexa Fluor™ 488 Conjugates) | Invitrogen™ | Cat#w11261 |
| Anti-Bcl2 (124) | Cell signaling | Cat#15071s |
| Anti-Mcl1 (D2W9E) | Cell signaling | Cat# 94296 |
| Anti-SOD2 (B-1) | Santa Cruz | Cat#sc-133254 |
| Anti-p16 (EPR1473) | Abcam | Cat#ab108349 |
| Isolectin IB4 Conjugates | Thermo Fisher | Cat# I21411 |
| Anti-MYH6 (K13) | Santa Cruz | Cat# sc-168676 |
| Anti-Human Nuclei Antibody (HNA, clone 3E1.3) | Millipore Sigma | Cat#MAB4383 |
| **Chemicals, Peptides, and Recombinant Proteins** | | |
| Human erythropotein | Sigma | Cat#E5627 |
| Human FGF | PeproTech | Cat#100-18B |
| FBS | Hyclone | Cat#SH30910.03 |

| | | |
|---|---|---|
| Penicillin/streptomycin | Gibco | Cat#15140122 |
| Glutathione | Sigma | Cat#G6013 |
| 5-Azacytidine | Sigma | Cat#A2385 |
| RPMI 1640 | Gibco | Cat#22400089 |
| B27® supplement with insulin free | Invitrogen | Cat#A1895601 |
| Trichostatin A | Sigma | Cat#T8552 |
| CHIR99021 | EMD Millipore | Cat#361571 |
| KY02111 | Sigma | Cat#SML0948 |
| XAV939 | Sigma | Cat#x3004 |
| LIF | PeproTech | Cat#300-05 |
| TGFb1 | Sigma | Cat#H8541 |
| Insulin | Sigma | Cat#I3536 |
| NRG-1 | R&D systems | Cat#396-HB |
| G-CSF | R&D systems | Cat#214-CS |
| Vitamin C | Sigma | Cat#A92902 |
| DMEM | Gibco | Cat#11995-065 |
| Matrigel | BD Biosciences | Cat#356234 |
| VEGF | PeproTech | Cat#100-20 |
| Ham's F-12 medium | Invitrogen | Cat#12-615F |
| PDGF-BB | PeproTech | Cat#100-14B |
| Smooth muscle cell differentiation medium | Sigma | Cat#311D-250 |
| Tetramethyl rhodamine methylester | Sigma | Cat#T5428 |
| Gelatin | Sigma | Cat# G9391 |
| 5-Bromo-2′-deoxyuridine | Sigma | Cat#B5002 |
| Tert-Butyl hydroperoxide solution | Sigma | Cat#B2633 |
| Hydrogen peroxide solution | Sigma | Cat#216763 |
| Dexamethasone | Sigma | Cat# D1756-1G |
| Cadmium chloride | Sigma | Cat#439800 |
| **Critical Commercial Assays** | | |
| AurumTM Total RNA mini kit | Bio-Rad | Cat#732-6820 |
| Bio-Rad iScriptTM cDNA Synthesis kit | Bio-Rad | Cat#170-8891 |
| iTaqTM Universal SYBR Green Supermix | Bio-Rad | Cat#172-5124 |
| Cellular ROS Detection Assay Kit | Abcam | Cat#ab113851 |
| SuperSignal™ West Pico PLUS Chemiluminescent Substrate | ThermoFisher | Cat#34577 |
| TUNEL Andy Fluor™ 488 Apoptosis Detection Kit | GeneCopoeia | Cat#A050 |
| Human Cytokine antibody library kit | RayBiotech | Cat# AAH-CYT-6-2 |
| Annexin V/Dead cell apoptosis kit | Invitrogen | Cat# V13241 |
| Human Cytokine Primer Library I | RealTimePrimers.com | Cat# HCA-I |
| Human Cytokine Primer Library II | RealTimePrimers.com | Cat# HCA-II |
| Thermo Scientific™ Richard-Allan Scientific™ Masson Trichrome Kit | Thermo Fisher Scientific | Cat# 22-110-648 |
| **Experimental Models: Cell Lines** | | |

| | | |
|---|---|---|
| $\Delta\psi_m^{low}$-hMPCs cell line | Division of Cardiothoracic Surgery, Albany Medical Center | Isolated from human left atrial appendages |
| $\Delta\psi_m^{high}$-RAACs cell line | Division of Cardiothoracic Surgery, Albany Medical Center | Isolation from human left atrial appendages |
| RAACs cell line | Division of Cardiothoracic Surgery, Albany Medical Center | Isolation from human left atrial appendages |
| **Experimental Models: Organisms/Strains** | | |
| NSG mouse model | The Jackson Laboratory | NOD.Cg-*Prkdc^scid^ Il2rg^tm1Wjl^*/SzJ |
| **Oligonucleotides** | | |
| See primer sequence for RT-PCR in Supplementary Table 5 | | |
| **Software and Algorithms** | | |
| GraphPad Prism 5 software | Prism | NA |
| Image J software | NIH | NA |
| Guava EasyCyte™ System software 3.1 | EMD Millipore | NA |
| Leica TCS SPE confocal microscope software | Leica | NA |
| NIS-elements AR version 5.0 | Nikon | NA |
| Bio-Rad CFX Manager software 3.0 | BioRad | NA |
| Vevo 3100 workstation software | FUJIFILM Visual Sonics | NA |
