# Supplementary material for: Mitochondrial Membrane Potential Identifies a Subpopulation of Mesenchymal Progenitor Cells to Promote Angiogenesis and Myocardial Repair
Source: Cells. 2022 May 22;11(10):1713. doi: 10.3390/cells11101713 (PMC9139404; doi:10.3390/cells11101713)
Supplement: Supplementary file 1 [file cells-11-01713-s001.zip › Supplementary Material-Detailed Methods.pdf]

Detailed Experimental Methods.

Human heart tissue collection and $\Delta\psi_m^{low}$-hMPCs isolation

The protocol for collecting human samples in this study were approved by the Institutional Committee on Research in Albany Medical College (IRB#3728), and written informed consent was signed by the patients. Human right atrial appendages (RAA) tissues (age from 42 to 74 years old) were collected and enzymatically digested for the establishment of human cardiac cell lines and defined as RAACs (Supplementary Table S1). We sorted human cardiac cells based on mitochondrial membrane potential ($\Delta\psi_m$) and collected ~3% cells with lowest $\Delta\psi_m$ that named as $\Delta\psi_m^{low}$-hMPCs (abbreviated as hMPCs in the manuscript). Briefly, after washing with cold PBS, the fresh human right atrial appendages were minced into small clumps (about 1-2mm^3 each) and incubated with 500 Units/ml collagenase II (Worthington Biochemical Cat# LS004177) at 37°C water bath for 1.5 hours. Chopped tissues were mechanically disturbed by pipette every 20 min. After incubation, separated undigested tissues by gravity on ice for 10 minutes, and transferred the supernatant to a new 50 ml tube. The dissociated cells were collected by centrifuging at 300 x g for 5 min. The cell pellet was resuspended and cultured in the growth medium containing Ham's F-12 medium (Invitrogen Cat#12-615F), 10% FBS (Hyclone Cat#SH30910.03), 0.005 Units/ml human erythropoietin (Sigma Cat#E5627), 10ng/ml human FGF (PeproTech Cat#100-18B), 100 Units/ml penicillin/streptomycin (Gibco Cat#15140122) and 0.2 mM glutathione (Sigma Cat#G6013). RAACs were maintained in a humidified incubate at 37°C, 5% CO_2 and 5% O_2. Replace the medium with fresh growth medium after 2 days of cell isolation and change the culture medium every 3 days. A total of 10^7 of RAACs were used to sort the human cardiac progenitor cells by LSRII flow cytometry (BD Biosciences) after incubating with 25 nM Tetramethylrhodamine methyl ester perchlorate (TMRM) red dye (a fluorescent dye for measuring mitochondrial membrane potential) for 30 min at 37 ℃ (Sigma Cat#T5428). We collected about ~3% of cells with the lowest $\Delta\psi_m$ and named as $\Delta\psi_m^{low}$-hMPCs, and 3% of cells at the highest $\Delta\psi_m$ as control cells. Passage 3 to 10 of $\Delta\psi_m^{low}$-hMPCs were used for in vitro experiments, and passage 4 to 6 of $\Delta\psi_m^{low}$-hMPCs used for in vivo experiments.

FACS analysis of cell surface markers for hMPCs

To characterize the cells based on the expression of cell surface markers, hMPCs were detached with 0.25% Trypsin-EDTA solution, washed 1 time with PBS and incubated with antibodies for different cell surface markers (see Supplementary Table S6, all diluted at 1:10 in the volume of 0.2 mL per 0.4 million) at 4 °C for 10 min. FACS analysis was performed by the Guava EasyCyte™ System. Cells followed with cardiac differentiation were dissociated and fixed with 4% PFA, then incubated with antibodies (1:200, FITC anti-cTNT, Abcam#ab8295; 1:200, Anti-Nkx2.5, Abcam #ab91196; 1: 200) overnight at 4 °C, and then incubated with second antibody (1:500) and subjected to the FACS analysis by Guava EasyCyte™ System (EMD Millipore Corporation, Inc). Detailed information for antibodies were listed in Supplementary Table S6.

RNA extraction and quantitative real-time polymerase chain reaction (qRT-PCR)

Real-time PCR was be performed to determine the effect of differentiation of cardiomyocytes, human cytokines library I & II screening (Supplementary Table S2 and S3) and other relative genes expression of

RAACs and hMPCs. The total RNA of each sample was be extracted using the AurumTM Total RNA minikit (Bio-Rad Cat#732-6820). The quality and quantity of RNA were detected by a NanoDrop 2000C spectrophotometer (Thermo Scientific). The reverse transcription of 1ug of RNA to cDNA was done by using the Bio-Rad iScriptTM cDNA Synthesis kit (Bio-Rad Cat#170-8891). Samples for real-time qPCR were prepared according to the manufacturer's instructions in the iTaqTM Universal SYBR Green Supermix (Bio-Rad Cat#172-5124), and was run with a Bio-Rad CFX384TM Real-Time system. The cycling conditions were set as below: 95°C for 3 min for initial denaturation, 45 cycles of denaturation at 95°C for 10s, and annealing/extension at 60°C for 30s. Melt curve analysis was set between 70 and 95 °C with 0.5 °C increments at 5s per step. In these experiments, GAPDH was used as the internal control gene for quantitative gene expression analysis. Primers were order from Integrated DNA Tech (IDT). The primer sequences were showed in the Supplementary Table S5.

In vitro cardiac lineage cell differentiation from hMPCs

A) Endothelial cell differentiation

hMPCs were plated (about 80% confluence) for 24 hours before performed endothelial cells differentiation. The protocol for endothelia cell differentiation was followed as reported by Dr. Oswald's, et al. [1]. Briefly, the culture medium was replaced with endothelial cells differentiated medium containing Ham's F-12 medium, 2% FBS (Hyclone Cat#SH30910.03), 50ng/ml VEGF (PeproTech Cat#100-20) and 1x Penicillin/Streptomycin. Endothelial differentiation medium was changed every 2 days. At 14 days, differentiated cells were collected and subjected to the immunofluorescence staining and FACS analysis with endothelial marker Von Willebrand factor (1:400, Sigma, Cat#F3520) to assess the efficiency of endothelial differentiation.

B) Smooth muscle cells (SMC) differentiation

hMPCs were plated (about 80% confluence) for 24 hours before performed SMC differentiation. The differentiation procedures were exactly followed according to the manufacturer's instructions from manufacture. Briefly, hMPCs were cultured in smooth muscle cells differentiation medium (Sigma Cat#311D-250) with 20ng/ml PDGF-BB (PeproTech Cat#100-14B), and medium was changed every 2 days. At day10, collected cells for immunofluorescence staining and FACS analysis for the SMC marker α-Smooth Muscle Actin (1:400, Sigma Cat#A2547) in order to evaluate the efficiency of smooth muscle differentiation.

Cellular ROS and TMRM measurement

Cellular ROS production was measured using a Cellular ROS Detection Assay Kit (Abcam Cat#ab113851). The experimental procedures were exactly followed according to manufacture instructions. hMPCs and RAACs were detached by 0.25% Trypsin-EDTA solution, and cell were collected and stressed with 500uM TBHP in FBS free medium for 1h. Cells were washed with PBS for one time, and resuspended with 100ul assay buffer including 1x ROS Deep Red Dye and incubated in 5% CO_2, 37°C for 30 min. The intensity of red fluorescence was detected by Guava EasyCyte™ System. The protocol for mitochondrial membrane potential measurement was exactly followed as described by Sukumar M, et al.[2] Mitochondrial membrane potential was detected using potentiometric dye of tetramethyl rhodamine methylester (TMRM) with final

concentration of 25nM, incubated for 30 min at 37°C. The intensity of TMRM fluorescence was measured by Guava EasyCyte™ System.

Immunoblotting

Western blotting analysis was performed according to a protocol as described previously[3] . The chemiluminescent signals were detected using SuperSignal™ West Pico PLUS Chemiluminescent Substrate (ThermoFisher) and imaged by the Image Quant LAS 4000 system. The primary antibodies used in this study were listed in the Supplementary Table S6. The secreted cytokines from hMPCs and RAACs were evaluated by the human cytokine antibody library (RayBiotech Cat# AAH-CYT-6-2) (Supplementary Table S4). Briefly, the serum-free cell culture medium for hMPCs and RAACs were collected after seeding cells for 48h. The procedure for antibody array were conducted according to manufacture instructions. For all western blotting, α-tubulin or GAPDH used as internal control protein to indicate the equal protein loading. Band density analysis was performed by Image J software. The primary antibodies were listed in Supplementary Table S6.

Cellular apoptosis assay

Cell apoptosis was investigated by dual staining with Alexa Fluor 488-Annexin V and Propidium Iodide (PI) (Invitrogen Cat# V13241). Cell apoptosis assay was performed according to the manufacture protocol as described previously[3] . To check effects of conditional medium on cell apoptosis, we seed hMPCs and RAACs in 10cm dishes about 70% confluence and collected the culture medium after 48 hours. Then collected conditional medium were added into a new 6 well plate that RAACs were plated, and culture RAACs with conditional medium for another 48 hours. Cells were then detached by 0.25% Trypsin-EDTA solution and collected for the further treatment with 2mM H_2O_2 in cell culture medium for 90 min, and Annexin V & PI staining were performed for FACS analysis. Data was analyzed by GuavaSoft™ Module software (EMD Millipore Corporation, Inc). The four quadrants (Q1, Q2, Q3, and Q4) are marked in all FASC analysis. Cells positive for Annexin V only (Q4), or double positive for both Annexin V and PI (Q2) were defined as apoptotic cells (Q2+Q4); cells positive for PI only (Q1) were defined as necrotic cells; and cells negative for both Annexin V and PI (Q3) were defined as live cells.

Proliferation assessment with BrdU

To examine the proliferation of hMPCs and RAACs, FACS analysis with BrdU staining was conducted. Briefly, hMPCs and RAACs were seeded into 60 mm dishes with 70% confluence, after 24 hours, replaced the medium with fresh growth medium supplemented with 10 μM BrdU and cultured for another 12 hours, cells were collected and fixed with 4% PFA in PBS for 15 min at room temperature. Then, cells were treated with 2M HCl for 30 min at room temperature and permeabilized by 0.5% Triton X-100 for another 10 min. After washing three times with PBS, cells were stained with BrdU antibody (1:500, Cell signaling, Cat#5292s) for 2 hours at room temperature in PBS containing 1% BSA, and then cell were washed for 3 times with PBS, followed by the incubation a secondary antibody (1: 500, Alexa Fluo®-488, Life Technologies Cat# A11001) for 1 hour at room temperature and washed for 3 times with PBS for FACS analysis.

Murine model of acute myocardial infarction and intramyocardial cell delivery

The in vivo experiments were performed following the National Institutes of Health Guidelines on the Use of Laboratory Animals and were approved by the Albany Medical College Committee on Animal Care. Breeding pairs of *NOD.Cg-Prkdc^{scid} Il2rg^{tm1Wjl}/SzJ* (NSG) male and female mice (age 10-12 weeks) were purchased from The Jackson Laboratory (Bar Harbor, ME). All mice were maintained in micro-isolator cages under specific pathogen-free conditions in a room with a temperature of 24℃, 55–65% relative humidity, and a 12-h light-dark cycle. Mice were anesthetized with 2% isoflurane inhalation with an isoflurane delivery system (Viking Medical, Medford, NJ), and then fixed on the operating pad in the right lateral decubitus position by taping all four extremities. Following the endotracheal intubation technique with a modified 22-gauge plastic cannula, mice were mechanically ventilated by connected to a mouse ventilator (Harvard apparatus, Holliston, MA) which supplied 2% isoflurane in oxygen (tidal volume 0.2 ml/min, rate 120 strokes/min). Rectal temperature was continuously monitored and maintained within 36-37°C using a heat pad. The chest was opened through a left-sided thoracotomy at the 4th intercostal space, and the tissue and muscle were dissected carefully. After removing the pericardium, a slipknot was tied around the left anterior descending (LAD) coronary artery 2 to 3 mm from its origin with a 6-0 silk suture (Ethicon K801) to induce myocardial ischemia. The ligation was deemed successful when the anterior wall of the left ventricle (LV) turned pale. After 60 minutes of ischemia, the slipknot was released, and the myocardium began reperfusion by observing a return of the pink-red color of the anterior wall of the LV after 15-20 sec. The procedure for human cells transplantation by intramyocardial injection was similar to that used in our previous studies of cell transplantation [4]. Briefly, at 45 minutes after reperfusion, RAACs, hMPCs (5×10^5 cells in 40 μl) or an equivalent volume of vehicle were injected intramyocardially using a 30-gauge needle. A total of four injections (10 μl each) were made in the per-infarct region in a circular pattern, at the border between infarcted and non-infarcted myocardium (Supplementary Fig. S3a). Two steps were performed to prevent back leak from intramyocardial injection: (1) after each injection, the needle was kept steady in the injection site for a few seconds before being withdrawn; (2) a cotton tip was gently pushed on the injection point while the needle was withdrawn and a few seconds after withdrawing the needle. The chest wall was closed, and the muscles returned back to their original position and the skin closed with 4-0 silk suture. The animal was gently disconnected from the ventilator and spontaneous breathing was seen immediately. Mice were then allowed to recover and monitored for 39 days until euthanasia and tissue collection. In order to examine the proliferation of endogenous cardiomyocytes, animals were intraperitoneally injected with BrdU (100 mg/kg body weight) once daily for up to 5 weeks post MI. In a subset of experiments, mice did not receive any BrdU administration.

Echocardiographic analysis

Serial M-mode echocardiographic images of mice subjected to 1-2% isoflurane anesthesia were obtained via Vevo 3100 imaging platform (Visual Sonics, Inc. Canada) at baseline (7 days prior to coronary occlusion/reperfusion and cell transplantation), 5 days after human cells treatment, and 35 days after cells transplantation (4 days prior to hemodynamic studies and euthanasia). Using a rectal temperature probe, body temperature was carefully maintained between 36.7 and 37.3 ˚C and maintained mice heart rate around 420 beats/min throughout the study (Supplementary Fig. S3b, c). Hearts were imaged in the short axis between the two papillary muscles. Each measurement was obtained with M-mode by averaging results from three consecutive heart beats. LV internal dimensions (LVID) at diastole and systole (LVIDd and LVIDs) were measured. LV ejection fraction (EF) was automatically calculated by the

echocardiography software using the formulas: EF (%) = 100 × [(LVIDd3–LVIDs)3)/ LVIDd3]. Digital images were analyzed off-line by blinded observers using the Vevo 3100 workstation software.

Hemodynamic analysis

At Day 39, in vivo cardiac hemodynamic function was evaluated just before euthanasia utilizing the Mikro-Tip® Pressure Volume System (MPVS) Ultra Foundation Systems (AD instruments, Australia) with 1.0 French PVR-1045 micro-tip ultra-miniature pressure–volume (PV) catheter (Millar, Houston, TX). Mice were anesthetized with 1-2% isoflurane, intubated and ventilated with a positive pressure ventilator (Harvard apparatus Minivent Hugo Sachs Electronik, ventilation rate 105/min, tidal volume 10.3 μl/g). Rectal temperature was kept between 36.7 and 37.3 °C. The right common carotid artery was isolated and cannulated (PV catheter) for determination of LV performance. The raw pressure and volume data collected in text files by the MPCU-200 unit and Chart/Powerlab software were imported into the PVAN software, which applied a variety of algorithms to the P-V data to calculate up to 30 cardiovascular parameters. As in the case of the echo study, all hemodynamic data analyses were performed off-line by investigators blinded to the treatment.

Heart fixation and morphometric analysis

At the end of the protocol, the heart was arrested in diastole by an *i.v.* injection of 0.15 ml of CdCl2 (100 mM), excised, and perfused retrogradely at 60–80 mmHg (LVEDP = 8 mmHg) with heparinized PBS followed by 10% neutral buffered formalin solution for 15 min. The heart was then sectioned into three slices from apex to base, fixed in formalin for 24 h, and subjected to tissue processing and paraffin embedding. Paraffin-embedded LV blocks were sectioned at a thickness of 4 μm for histochemistry and immunohistochemistry. To evaluate cardiac interstitial fibrosis and structural changes, three sections (4 μm thick) per heart were prepared for Masson's trichrome staining per manufacturer's instructions (Thermo Scientific™, Richard-Allan Scientific™ Masson Trichrome Kit). The stained sections were examined under a light microscope (Leica ICC50 HD), and the morphometric parameters, including total LV area, risk region area, scar area, and infarct expansion index were measured by NIH Image J (1.42v) software.

Immunohistochemistry staining

Paraffin-embedded, 5 μm thick heart sections were deparaffinized in xylene and rehydrated gradually through 100, 95, and 70% ethanol followed by antigen retrieval procedure. After pre-incubation with 5% goat serum blocking solution, the primary antibodies were applied to identify the expression of different proteins, including anti-Human Nuclei antibody (1:100, HNA, Milipore, CA) for the newly formed cardiac cells derived from transplanted human cells, anti-BrdU antibody for newly formed cells (1:500, Abcam, MA), anti-α Smooth Muscle Actin (1:500, SMA) antibody (Sigma, MO) for vessels, anti-von Willebrand Factor (vWF) antibody (1:300, Sigma, MO) for endothelial cells, anti-α Sarcomeric Actin (α-SA) antibody (1:500, Sigma, MO) for cardiomyocytes. Isolectin GS-IB4 conjugated with Alex Fluor 488 was used to stain small vessels. In all of the immunofluorescence staining procedures, secondary antibodies conjugated with the appropriate fluorochrome (Invitrogen, CA) were used, and nuclei were counterstained with DAPI (1/5000). The concentration of primary and secondary antibodies was used by following the manufacturer suggestions. To minimize the auto fluorescence, slides were incubated with 0.1% Sudan Black B (Sigma, MO), and washed with PBS for three times, and then mounted with ProLong Gold antifade reagent

(Invitrogen, CA). Immunohistochemically signals were imaged by Leica confocal laser-scanning microscope (Leica DMI 4000B) and quantitatively analyzed by Image J software. In all cases, at least six hearts per group were examined. In each heart, we cut serial LV sections and take three sections for the quantitative analysis at ~100-120 μm intervals, which are below the ligation site and along the LV longitudinal axis.

TUNEL assay for assessment of apoptotic cell death

Terminal deoxynucleotidyltransferase-mediated dUTP nick-end labeling (TUNEL) was performed to detect apoptotic nuclei by using terminal deoxynucleotidyltransferase-mediated in situ fluorescein-conjugated, dUTP nick end-labeling technique according to the manufacturer's protocol (GeneCopoeia, Inc. MD). The sections were incubated with mouse monoclonal antibody against α-SA (Sigma, MO) to identify cardiomyocytes, and nuclei were counterstained with DAPI (1/5000). The fluorescence staining was viewed with Leica confocal laser-scanning microscope. The number of apoptotic cells with TUNEL-positive nuclei was expressed as a percentage of total cell population.

Wheat Germ Agglutinin staining

To evaluate the cardiac fibrosis after myocardial infarction with or without cell transplantation, wheat germ agglutinin (WGA) (Invitrogen) staining was performed for heart tissue sections among three groups. The procedures were exactly followed according to manufacture instructions. Briefly, WGA was diluted in HBSS buffer at the final concentration of 10μg/ml as work solution. Heart sections were deparaffinized in xylene and rehydrated gradually through 100, 95, and 70% ethanol followed by antigen retrieval procedure. Deparaffinized sections were incubated with WGA work solution in dark box for 1 hour at 37°C. Then the slides were washed with PBS for 3 times. The whole heart scanning was performed with Nikon confocal laser-scanning microscope (Nikon Eslipse Ti2).

Reference

1. Oswald J, Boxberger S, Jorgensen B, Feldmann S, Ehninger G, Bornhauser M, Werner C. Mesenchymal stem cells can be differentiated into endothelial cells in vitro. *Stem cells*. 2004;22:377-384
2. Sukumar M, Liu J, Mehta GU, Patel SJ, Roychoudhuri R, Crompton JG, Klebanoff CA, Ji Y, Li P, Yu Z, Whitehill GD, Clever D, Eil RL, Palmer DC, Mitra S, Rao M, Keyvanfar K, Schrump DS, Wang E, Marincola FM, Gattinoni L, Leonard WJ, Muranski P, Finkel T, Restifo NP. Mitochondrial membrane potential identifies cells with enhanced stemness for cellular therapy. *Cell metabolism*. 2016;23:63-76
3. Li X, He P, Wang XL, Zhang S, Devejian N, Bennett E, Cai C. Sulfiredoxin-1 enhances cardiac progenitor cell survival against oxidative stress via the upregulation of the erk/nrf2 signal pathway. *Free radical biology & medicine*. 2018;123:8-19
4. Cai C, Guo Y, Teng L, Nong Y, Tan M, Book MJ, Zhu X, Wang XL, Du J, Wu WJ, Xie W, Hong KU, Li Q, Bolli R. Preconditioning human cardiac stem cells with an ho-1 inducer exerts beneficial effects after cell transplantation in the infarcted murine heart. *Stem Cells*. 2015;33:3596-3607
